# Analysis of relative error in perturbation Monte Carlo simulations of radiative transport

**DOI:** 10.1117/1.JBO.28.6.065001

**Published:** 2023-06-07

**Authors:** Mahsa Parsanasab, Carole Hayakawa, Jerome Spanier, Yanning Shen, Vasan Venugopalan

**Affiliations:** a University of California, Irvine, Department of Chemical and Biomolecular Engineering, Irvine, California, United States; b University of California, Irvine, Beckman Laser Institute and Medical Clinic, Irvine, California, United States; c University of California, Irvine, Department of Electrical Engineering and Computer Science, Irvine, California, United States

**Keywords:** Monte Carlo simulation, perturbation methods, radiative transport, inverse problems, uncertainty estimation, multispectral analysis, optical imaging

## Abstract

**Significance:**

Perturbation and differential Monte Carlo (pMC/dMC) methods, used in conjunction with nonlinear optimization methods, have been successfully applied to solve inverse problems in diffuse optics. Application of pMC to systems over a large range of optical properties requires optimal “placement” of baseline conventional Monte Carlo (cMC) simulations to minimize the pMC variance. The inability to predict the growth in pMC solution uncertainty with perturbation size limits the application of pMC, especially for multispectral datasets where the variation of optical properties can be substantial.

**Aim:**

We aim to predict the variation of pMC variance with perturbation size without explicit computation of perturbed photon weights. Our proposed method can be used to determine the range of optical properties over which pMC predictions provide sufficient accuracy. This method can be used to specify the optical properties for the reference cMC simulations that pMC utilizes to provide accurate predictions over a desired optical property range.

**Approach:**

We utilize a conventional error propagation methodology to calculate changes in pMC relative error for Monte Carlo simulations. We demonstrate this methodology for spatially resolved diffuse reflectance measurements with ±20% scattering perturbations. We examine the performance of our method for reference simulations spanning a broad range of optical properties relevant for diffuse optical imaging of biological tissues. Our predictions are computed using the variance, covariance, and skewness of the photon weight, path length, and collision distributions generated by the reference simulation.

**Results:**

We find that our methodology performs best when used in conjunction with reference cMC simulations that utilize Russian Roulette (RR) method. Specifically, we demonstrate that for a proximal detector placed immediately adjacent to the source, we can estimate the pMC relative error within 5% of the true value for scattering perturbations in the range of [−15%,+20%]. For a distal detector placed at ∼3 transport mean free paths relative to the source, our method provides relative error estimates within 20% for scattering perturbations in the range of [−8%,+15%]. Moreover, reference simulations performed at lower $\left(\mu_{s}^{\prime }/\mu_{a}\right)$ values showed better performance for both proximal and distal detectors.

**Conclusions:**

These findings indicate that reference simulations utilizing continuous absorption weighting (CAW) with the Russian Roulette method and executed using optical properties with a low $\left(\mu_{s}^{\prime }/\mu_{a}\right)$ ratio spanning the desired range of $\mu_{s}$ values, are highly advantageous for the deployment of pMC to obtain radiative transport estimates over a wide range of optical properties.

## 1 Introduction

Monte Carlo simulations have been broadly adopted by the biomedical optics community to simulate light propagation in scattering tissues on mesoscopic and macroscopic scales; i.e., on spatial scales comparable to and larger than the single scattering mean free path. Conventional Monte Carlo (cMC) simulations provide rigorous solutions to the radiative transport equation and can be configured for systems with complicated geometric and material features. However, cMC simulations can be computationally costly since this stochastic solver carries with it a solution uncertainty that scales as $\frac{1}{\sqrt{N}}$ where N is the number of photons simulated.^1^ Thus, any application that requires the execution of multiple cMC simulations; e.g., the resolution of an inverse problem, can easily have an associated computational cost that is impractical given the number of photons that must be simulated in order to obtain an estimate with sufficiently low uncertainty.

The computational cost associated with Monte Carlo simulation has motivated many groups to develop methods to improve its speed and efficiency for simulations of light transport in turbid media.^2^ These methods can be generally categorized as follows: lookup table-based MC,^3^ scaled or “white” MC,^4^^–^^8^ perturbation MC,^9^ parallel computing, cloud computing and/or graphic processor unit (GPU)-based methods^10^^–^^12^ and variance reduction techniques.^13^ Amongst these, both lookup table and scaled methods suffer from restrictions to a fixed pre-defined geometry and prior binning of results that lead to reductions in accuracy.^7^ Parallel computing and GPU-based methods accelerate the speed of Monte Carlo simulations using innovations in compilers and hardware. However, the MC simulation engine remains unchanged, and often existing codes must be restructured to reap the benefits.

The perturbation Monte Carlo (pMC) method has been developed to rapidly obtain estimates for systems that are slightly modified, in terms of optical properties and/or geometry, relative to a reference cMC simulation. Moreover, the pMC framework facilitates implementation of a ‘sister’ method known as differential Monte Carlo (dMC) that enables the computation of sensitivities (Jacobian) for the resolution of inverse problems using gradient-based optimization methods.^14^^–^^16^

The pMC method leverages correlated sampling by using a single set of random walks for simultaneous analysis of a ‘reference’ system together with any number of closely related systems which are modified in terms of optical properties and/or geometric characteristics.^1^ pMC methods enable the rapid computation of RTE solutions for these closely related systems by post-processing the random walks from a database formed by characteristics of the reference MC simulation.^9^^,^^14^^,^^17^^–^^19^ In this database, the weight, path length, and the number of collisions experienced by each detected photon are stored. pMC analysis modifies the weight of each tallied photon in the reference database based on its path length and number of collisions and change of the optical properties relative to the reference system.^1^^,^^9^^,^^20^

Several studies have implemented pMC to improve computational efficiency and accuracy as compared to cMC simulations. Yamamoto and Sakamoto^21^ used pMC to reconstruct the optical characteristics of a heterogeneous, two-dimensional tissue model using temporal frequency domain data. This approach effectively reconstructed both scattering and absorption coefficients. For diffuse optical tomography applications, Yao et al.^22^ extended pMC to compute spatially and temporally resolved sensitivity profiles. A novel method for solving the inverse problem of quantitative photoacoustic tomography using pMC was provided in another paper by Leino et al.^16^ In this study, pMC was shown to be capable of estimating spatial distributions of both absorption and scattering parameters. These estimated distributions were quantitatively accurate over the full range of parameter values typical for biological tissues.

Despite these achievements, challenges remain to broadly apply pMC methods for the analysis of multispectral datasets. Prior studies have applied pMC to datasets acquired at a single or small number of wavelengths.^9^^,^^14^^,^^15^^,^^17^ Given the broad range of optical properties spanned in multispectral datasets, reference MC simulations for multiple sets of optical properties are likely needed. Yet, the specific optical property values that should be chosen to minimize the number of reference simulations required are unknown. While it is known that pMC uncertainty, and therefore accuracy, degrades with increases in perturbation size (degree of the tissue optical property change),^20^ a general method to quantify the growth in the pMC uncertainty with perturbation size has not been proposed. Currently, the accuracy of a pMC simulation can only be assessed after the pMC computation is performed. This is clearly undesirable since we would like to know *a priori*, how large a perturbation can be computed from a reference simulation before the accuracy of the resulting pMC estimate becomes unacceptable.

*A priori* quantitative prediction of the growth of pMC uncertainty with perturbation size using data from the reference simulation alone would facilitate the implementation of pMC/dMC methods. This is because once the reference simulation is performed the growth in pMC uncertainty with perturbation size could be quantified thereby identifying the range of optical properties for which the reference simulation could be utilized. This would facilitate the analysis of multispectral datasets where there are often large variations in optical absorption and scattering properties. Our first objective in this study is to identify the range of perturbation size that could be applied to a reference simulation while retaining the pMC estimate variance within a certain limit. To do so we determine the pMC estimate variance (which is directly related to the pMC estimate relative error) as a function of perturbation size and optical properties to identify an acceptable perturbation range for each reference simulation. We then develop a method for *a priori* prediction of pMC uncertainty using information from the reference simulation alone without explicitly computing the perturbed photon weights. This enables the prediction of the largest perturbation size for which pMC can still be used to provide an estimate with an acceptable relative error.

## 2 Method

We start by describing the formulation of pMC and its use to obtain a mean detected photon weight and associated variance which captures the uncertainty of the mean estimate. Rigorous computation of the variance associated with a pMC estimate is best obtained by analyzing the population of perturbed photon weights. These photon weights are determined by post-processing the database that stores the characteristics of each detected photon from the reference simulation. Although the photons perturbed weight variance can be accurately calculated for each perturbation size, our objective is to avoid such rigorous calculations and determine an accurate variance estimate for a range of perturbation sizes using only data from the reference simulation. This includes various order moments of weight, the number of collisions, and path length distributions, along with their corresponding covariance values.

We consider a pMC simulation of a homogeneous semi-infinite tissue whereby the optical properties of the perturbed system are changed globally. In pMC, the weight of only those photons that are tallied at the detector under consideration is modified. The perturbed photon weight for the i’th photon ($W_{P,i}$) with perturbed optical properties ($\mu_{a,P},\mu_{s,P}$) is computed from the i’th photon weight from the reference simulation ($W_{R,i}$), which utilized reference optical properties ($\mu_{a,R},\mu_{s,R}$) as follows:^9^

$$W_{P,i}=W_{R,i}{\left(\frac{\mu_{s,P}}{\mu_{s,R}}\right)}^{j_{i}}\;\exp[-(\mu_{t,P}-\mu_{t,R})L_{i}],$$ (1)

where $\mu_{s}$ and $\mu_{t}$ are the scattering and total interaction coefficients ($\mu_{t}=\mu_{a}+\mu_{s}$, $\mu_{a}$ being the absorption coefficient), respectively, $j_{i}$ is the number of collisions the i’th tallied photon experiences in the medium, and $L_{i}$ is the path length taken by the i’th photon prior to detection. The subscripts ‘P’ and ‘R’ refer to the perturbed and reference cases, respectively. To generate a set of perturbed photon weights, Eq. (1) is applied to each photon that is tallied at the detector in the reference simulation. The variance of the entire population of photon weights $\sigma_{W_{P}}^{2}$, for the perturbed case is given as

$$\sigma_{W_{P}}^{2}=\frac{1}{N}\overset{N}{\underset{i=1}{\sum }}{\left(W_{P,i}-{\overset{¯}{W}}_{P}\right)}^{2},$$ (2)

where N is the total number of photons launched and ${\overset{¯}{W}}_{P}$ is the mean weight of the population of photon weights for the perturbed case, $W_{P,i}$. Our goal is to estimate the variance of the perturbed photon weights from the reference simulation alone, i.e., without explicitly calculating the perturbed photon weights needed to apply Eq. (2).

The database obtained from the reference Monte Carlo simulation contains not only the weight of each photon but also the number of photon collisions and path length. Given that the photon weights, collisions, and path lengths in MC simulations of radiative transport are frequently not normally distributed,^23^ we will examine the utility of applying a classical error propagation approach inclusive of the second-order (covariance) and third-order (skewness) terms to estimate the variance associated with the mean perturbed photon weight. Application of this approach to Eq. (1) provides the following equation to estimate the variance of the population of perturbed photon weights $\sigma_{W_{P}}^{2}$:^24^

$$\sigma_{W_{P}}^{2}\approx {\left(\frac{\partial W_{P}}{\partial W_{R}}\right)}^{2}\sigma_{W_{R}}^{2}+{\left(\frac{\partial W_{P}}{\partial j}\right)}^{2}\sigma_{j}^{2}+{\left(\frac{\partial W_{P}}{\partial L}\right)}^{2}\sigma_{L}^{2}\;+2(\frac{\partial W_{P}}{\partial W_{R}})(\frac{\partial W_{P}}{\partial j})\sigma_{W_{R},j}+2(\frac{\partial W_{P}}{\partial W_{R}})(\frac{\partial W_{P}}{\partial L})\sigma_{W_{R},L}+2(\frac{\partial W_{P}}{\partial j})(\frac{\partial W_{P}}{\partial L})\sigma_{j,L}\;+(\frac{\partial W_{P}}{\partial W_{R}})(\frac{\partial^{2}W_{P}}{\partial W_{R}^{2}})\gamma_{W_{R}}\sigma_{W_{R}}^{3}+(\frac{\partial W_{P}}{\partial j})(\frac{\partial^{2}W_{P}}{\partial j^{2}})\gamma_{j}\sigma_{j}^{3}+(\frac{\partial W_{P}}{\partial L})(\frac{\partial^{2}W_{P}}{\partial L^{2}})\gamma_{L}\sigma_{L}^{3}.$$ (3)

In this equation $\sigma_{x}$ and $\sigma_{x}^{2}$ represent the standard deviation and variance of the random variable x, respectively, shown in Eq. (4a). $\sigma_{x,y}$ represents the covariance between the two random variables x and y shown in Eq. (4b). $\gamma_{x}$ provides a measure of skewness of the distribution of the random variable x,^25^ as defined by Eq. (4c). The product $\gamma_{x}\sigma_{x}^{3}$ represents the third moment of the random variable x:

$$\sigma_{x}^{2}=\frac{1}{N}\sum {\left(x_{i}-\overset{¯}{x}\right)}^{2}\to \sigma_{x}=\sqrt{\frac{1}{N}\sum {\left(x_{i}-\overset{¯}{x}\right)}^{2}},$$ (4a)

$$\sigma_{x,y}=\frac{1}{N}\sum (x_{i}-\overset{¯}{x})(y_{i}-\overset{¯}{y}),$$ (4b)

$$\gamma_{x}=\frac{1}{N}\sum {\left(\frac{x_{i}-\overset{¯}{x}}{\sigma_{x}}\right)}^{3}.$$ (4c)

In general, for a specific detector collecting photons over a finite interval of space, time, and/or propagation direction, only a subset of the N photons that are simulated are tallied at the detector ($N_{T}$) while the remaining photons ($N_{U}$) go untallied. We thus recast Eq. (2) defining the variance of the perturbed photon weights $W_{P}$, in a form that separates contributions of the tallied and untallied photons to the variance of the perturbed photon weight. As detailed in Section A of the Supplemental Materials, this reformulation results in the following expression for the variance of the perturbed estimate:

$$\sigma_{W_{P}}^{2}=\frac{1}{N}[N_{T}(\sigma_{W_{P,T}}^{2}+{\overset{¯}{\Phi }}^{2})+N_{U}{\overset{¯}{W}}_{P}^{2}],$$ (5)

where $\sigma_{W_{P,T}}^{2}$ is the variance of the sub-population of photon weights that are tallied at the detector. $\overset{¯}{\Phi }={\overset{¯}{W}}_{P}-{\overset{¯}{W}}_{P,T}$ represents the difference of the mean weight over the entire population of simulated photons (N) and only the population of photons that are tallied at the detector ($N_{T}$). In this restructured equation, the contribution of the untallied photon population to the overall variance is accounted for via the $\frac{N_{U}}{N}{\overset{¯}{W}}_{P}^{2}$ term and the impact of the tallied photons is expressed through the variance of the population of the tallied photon weights alone plus the correction factor $\frac{N_{T}}{N}{\overset{¯}{\Phi }}^{2}$ that accounts for the differing sizes of the tallied population and the entire population of simulated photons.

The nonlinearities inherent in Eq. (1) can lead to a large dynamic range of the perturbed photon weights. For this reason, we choose to apply Eq. (3) on the linearized form of Eq. (1) and estimate the variance of the natural logarithm of the photon weights, $\sigma_{\ln(W_{P,T})}^{2}$ as follows:

$$\ln(W_{P,i})=\ln(W_{R,i})+j_{i}\;\ln(\frac{\mu_{s,P}}{\mu_{s,R}})-(\mu_{t,P}-\mu_{t,R})L_{i}.$$ (6)

Once $\sigma_{\ln(W_{P,T})}^{2}$ has been calculated, we estimate $\sigma_{W_{P,T}}^{2}$ using:

$$\sigma_{\ln(W_{P,T})}^{2}\approx {\left[\frac{\partial (\ln\;W_{P,T})}{\partial W_{P,T}}\right]}^{2}\sigma_{W_{P,T}}^{2},$$ (7)

$$\sigma_{W_{P,T}}^{2}\approx {\overset{¯}{W}}_{P,T}^{2}\times \sigma_{\ln(W_{P,T})}^{2}.$$ (8)

Finally, throughout the estimation process, we replace ${\overset{¯}{W}}_{P}$ with ${\overset{¯}{W}}_{R}$ to eliminate the need to calculate the mean perturbed weight. This replacement is expected to provide a reasonable approximation for small perturbations.

**Fig. 1:**
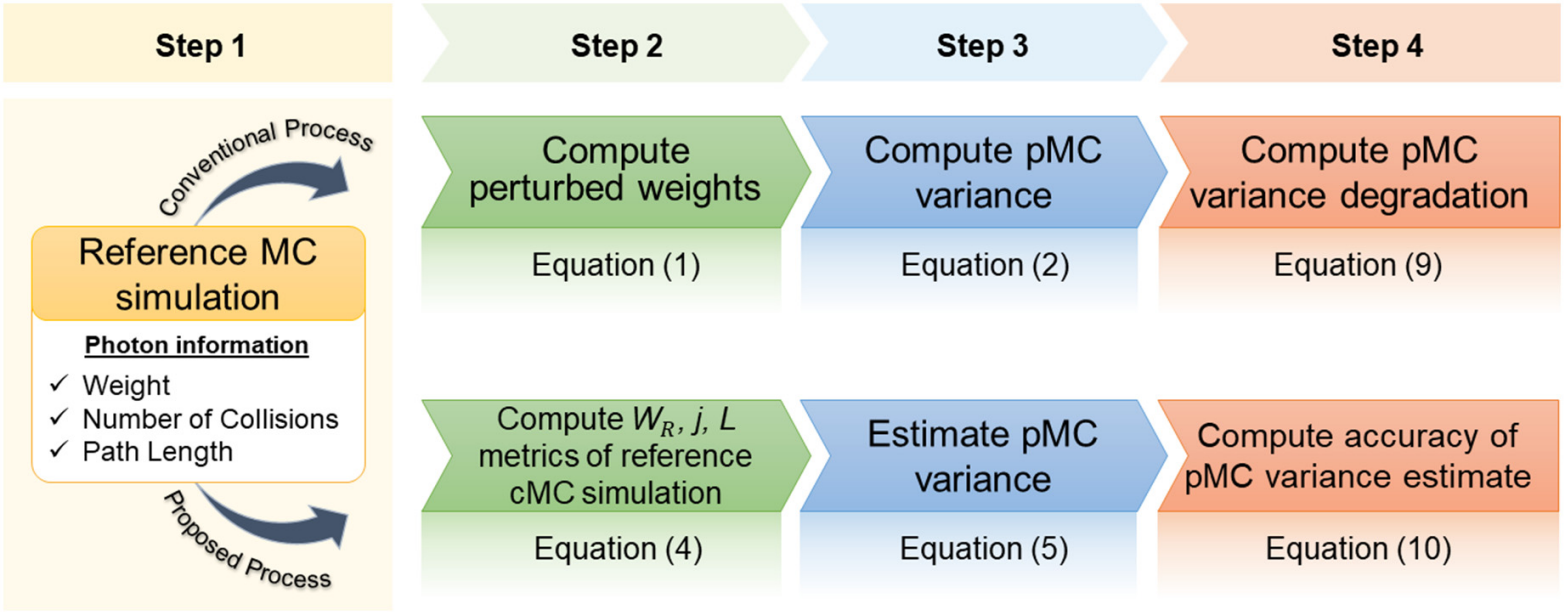
Schematic representation of the conventional variance calculation and our proposed variance estimation methods.

Figure 1 summarizes the conventional process to calculate the pMC variance along with our proposed process. The conventional process requires performing the pMC computation (steps 2 to 4) for each perturbation size of interest. By contrast, our proposed strategy computes statistical metrics that characterize the distributions of $W_{R}$, j, and L from the reference simulation alone (step 2) from which the pMC variance can be estimated (steps 3 and 4) for any perturbation size of interest. The equations characterizing the variation of pMC variance with perturbation size and the accuracy of our pMC variance estimate are presented in Eqs. (9) and (10).

## 3 Model Problem

To examine the performance of our method, we considered the case of spatially resolved reflectance in a homogeneous, semi-infinite medium. We performed conventional Monte Carlo simulations in which 20 million photons are launched from a directional point source in the reference simulations which utilize continuous absorption weighting (CAW).^23^ We consider a medium with fixed refractive index (n=1.4), single-scattering anisotropy (g=0.8), and transport mean free path ($l^{*}=1\;\mathrm{mm}$). We consider five different media with different ratios of reduced scattering to absorption coefficients $\left(\mu_{s}^{\prime }/\mu_{a}\right)$. Collection of photons happens at a proximal detector positioned immediately adjacent to the source spanning radial locations ρ∈[0 to 0.2]  mm and a distal detector spanning radial locations ρ∈[3 to 3.2]  mm. These two detectors are chosen to examine the characteristics of the pMC estimates as the collected signals at these two detectors have differing sensitivities to changes in optical absorption and scattering.^14^ As described below, we also performed cMC simulations utilizing the Russian Roulette method^26^ with a weight threshold of $10^{-3}$ and a survival probability of 0.1. Table 1 provides the optical properties for the five reference cases investigated.

**Table 1:** Optical parameters used for reference MC simulations with detectors placed at ρ∈[0 to 0.2] mm (proximal) and ρ∈[3 to 3.2]  mm (distal), g=0.8 was used in all simulations.

$\left(\mu_{s}^{\prime }/\mu_{a}\right)$	$\mu_{a}$ (/mm)	$\mu_{s}$ (/mm)	$\left(\mu_{s}/\mu_{t}\right)$
5	0.1667	4.1667	0.9615
10	0.0909	4.5455	0.9804
20	0.0476	4.7619	0.9901
50	0.0196	4.9020	0.9960
100	0.0099	4.9505	0.9980

The database resulting from each reference simulation was processed using pMC for scattering perturbations ($\epsilon_{s}$) over the range of [−20%,20%] in 5% increments and all other optical properties were left unchanged. We restricted our analysis to the consideration of scattering perturbations, since for conventional MC simulations utilizing CAW, perturbations in absorption can be accommodated without the loss of accuracy regardless of perturbation size.^7^

We introduce two metrics to characterize pMC performance and our ability to accurately calculate the pMC relative error using information from the reference MC simulation alone. First, we define a metric ${\overset{¯}{\Delta }}_{P}$ called the “degradation degree” that quantifies the relative error of a pMC estimate relative to the reference simulation. Computation of the variation of ${\overset{¯}{\Delta }}_{P}$ with perturbation size ($\epsilon_{s}$) enables the examination of the intrinsic accuracy of pMC. We also define δ which quantifies the relative difference between our estimate for the pMC relative error using information from the reference MC simulation alone compared with the actual pMC relative error. These two metrics are defined as follows:

$${\overset{¯}{\Delta }}_{P}=\frac{\sigma_{W_{P}}/{\overset{¯}{W}}_{P}}{\sigma_{W_{R}}/{\overset{¯}{W}}_{R}},$$ (9)

$$\delta =\frac{\sigma_{W_{P},\mathrm{est}}-\sigma_{W_{P}}}{\sigma_{W_{P}}},$$ (10)

where ${\overset{¯}{W}}_{R}$ and ${\overset{¯}{W}}_{P}$ are the mean photon weight from the reference Monte Carlo simulation and the pMC simulation, respectively.

## 4 Results and Discussions

**Fig. 2:**
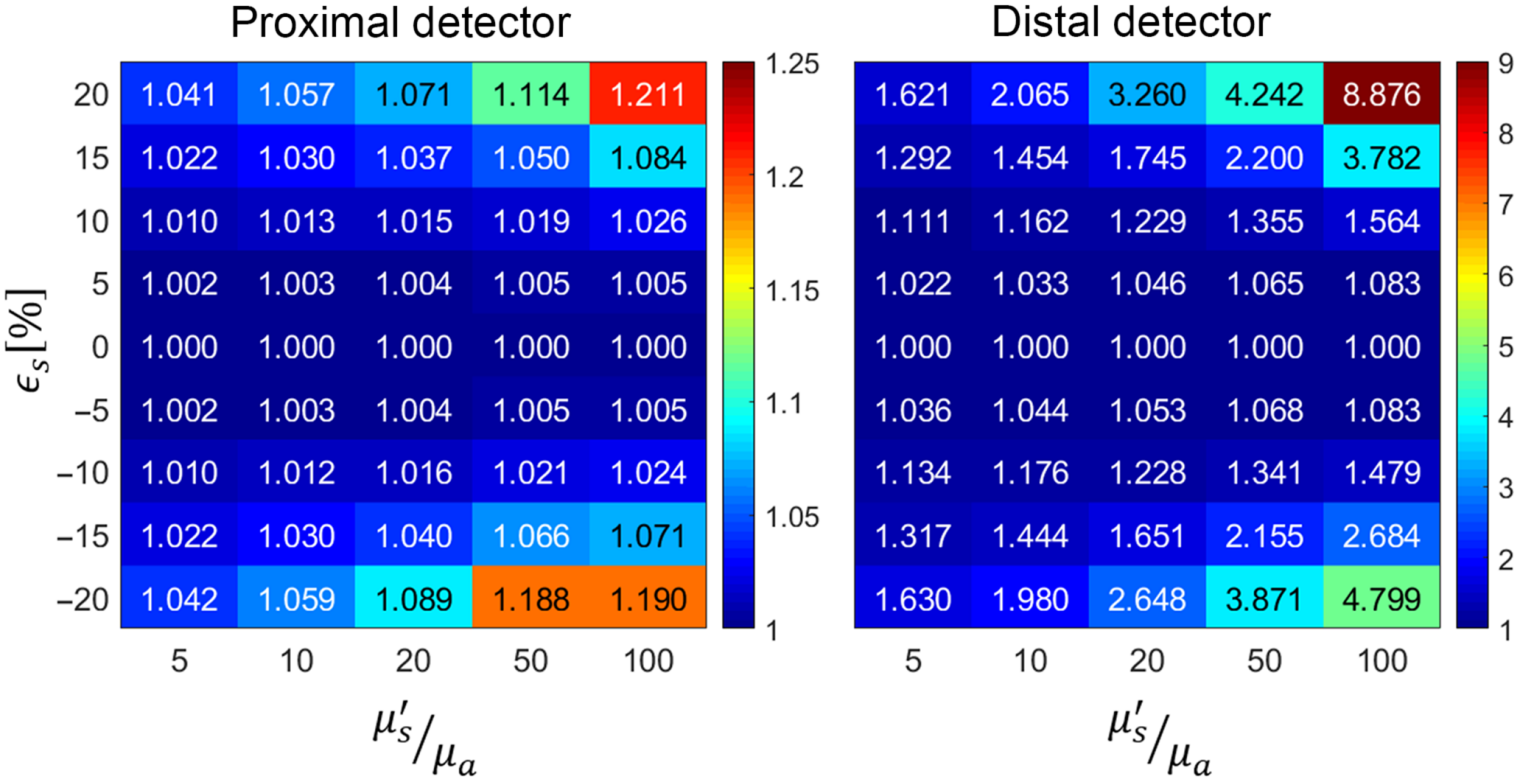
Variation of the degradation degree, ${\overset{¯}{\Delta }}_{P}$, with scattering perturbation size $\epsilon_{s}$ and optical properties $\left(\mu_{s}^{\prime }/\mu_{a}\right)$.

Figure 2 shows the variation in the degradation degree ${\overset{¯}{\Delta }}_{P}$ for the five sets of optical properties based on rigorous application of pMC calculations for $\epsilon_{s}$ range of [−20%,20%]. The mean and variance of all the pMC calculations are provided in Section B of the Supplemental Material. For the proximal detector, we see minimal changes in the pMC relative error as compared with the reference simulation, with only slight increases observed at scattering perturbations of ±20%. We do see, however, a slight worsening of accuracy for higher values of $\left(\mu_{s}^{\prime }/\mu_{a}\right)$. The situation is notably different for the distal detector with much more significant increases in relative error as compared with the reference simulations for both positive and negative scattering perturbations. Importantly, we see sharper escalations in pMC relative error for the simulations utilizing larger values of $\left(\mu_{s}^{\prime }/\mu_{a}\right)$. Also, in the $\epsilon_{s}$ range of [−10%,10%], we generally observe that for the reference simulations performed using larger $\left(\mu_{s}^{\prime }/\mu_{a}\right)$ values, the pMC relative error increases more sharply for positive scattering perturbations as compared with negative perturbations. Whereas for reference simulations performed at lower $\left(\mu_{s}^{\prime }/\mu_{a}\right)$ values, the pMC relative error degrades more sharply for negative perturbations.

These results indicate that the optical properties do not play as prominent role in pMC accuracy for the proximal detector as compared with the distal detector, where reference simulations performed at lower values of $\left(\mu_{s}^{\prime }/\mu_{a}\right)$ result in pMC predictions with much higher fidelity. Also, since the changes of ${\overset{¯}{\Delta }}_{P}$ relative to $\epsilon_{s}$ are asymmetric, it appears that positive scattering perturbations may be more robust in terms of reduced degradation in the pMC relative error for cases with lower $\left(\mu_{s}^{\prime }/\mu_{a}\right)$ whereas for higher $\left(\mu_{s}^{\prime }/\mu_{a}\right)$ increases in the degradation degree are smaller for negative scattering perturbations.

**Fig. 3:**
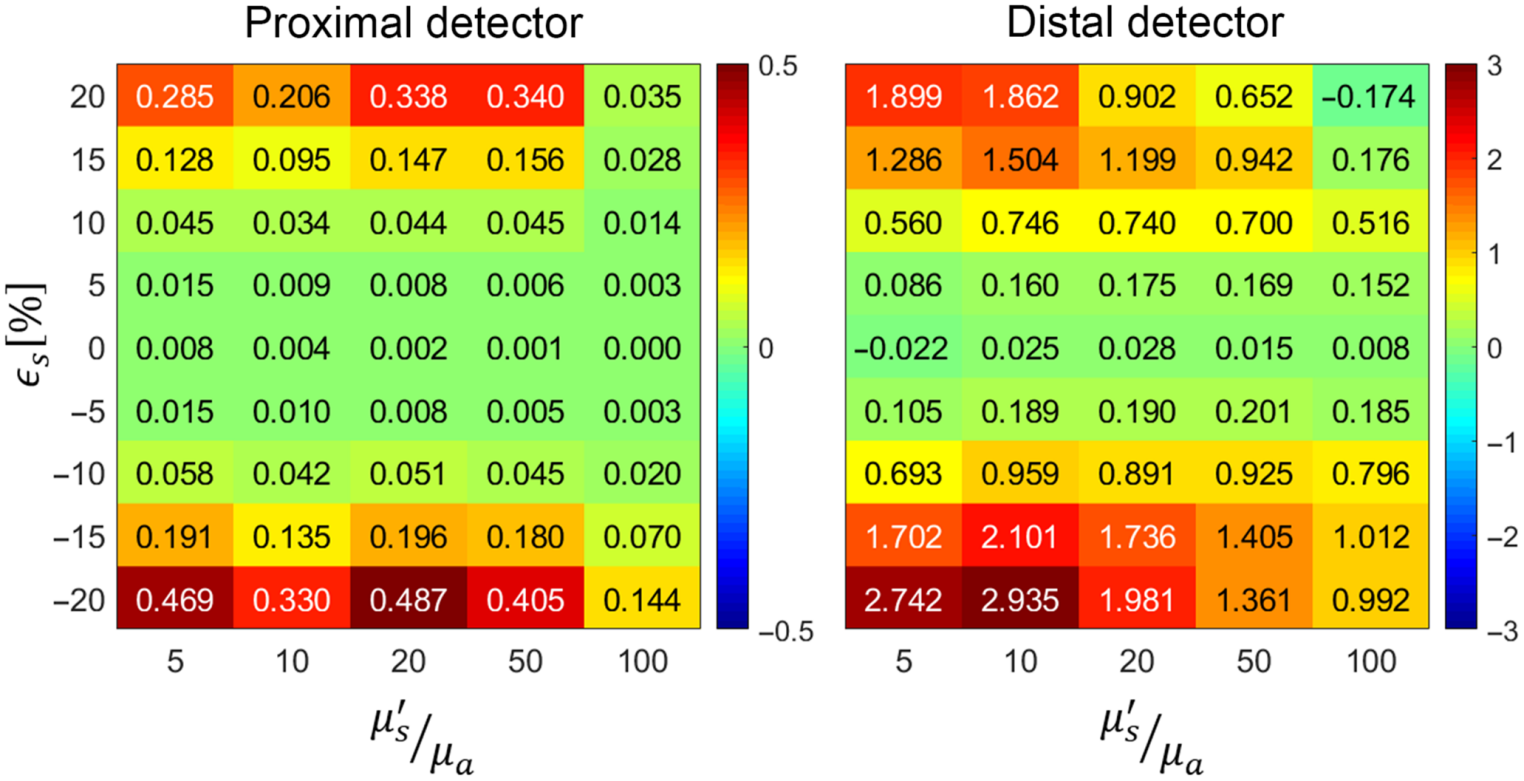
Variation of δ with scattering perturbation size $\epsilon_{s}$ and optical properties $\left(\mu_{s}^{\prime }/\mu_{a}\right)$.

To evaluate the accuracy in estimating the pMC relative error from the reference simulation as compared with a rigorous analysis obtained through conventional process of variance calculation, in Fig. 3 we display values for the δ metric for all the cases examined.

Our estimation of the pMC relative error based on distribution metrics of the reference simulation data alone incurs a relative error that generally grows with perturbation size for all reference cases. Our estimation method shows high fidelity in predicting the pMC relative error for the proximal detector over a substantial range of $\epsilon_{s}$ values. On the other hand, for the distal detector, the range of $\epsilon_{s}$ values that yield accurate estimates are confined to a much narrower range of $\epsilon_{s}$. Moreover, the growth in relative error is asymmetrical and our method provides better accuracy for positive scattering perturbations. Figure 3 also shows that our pMC error estimates are conservative and that we almost invariably provide overestimates of the relative error. In the case of the proximal detector, our method provides pMC relative error estimates within 10% of the true value for scattering perturbations in the range of $\epsilon_{s}=\pm 10\%$. The accuracy of our method for the distal detector is notably worse with error estimates within 15% for scattering perturbations in the range of $\epsilon_{s}=[-4\%,4\%]$ with much poorer performance outside this range.

**Fig. 4:**
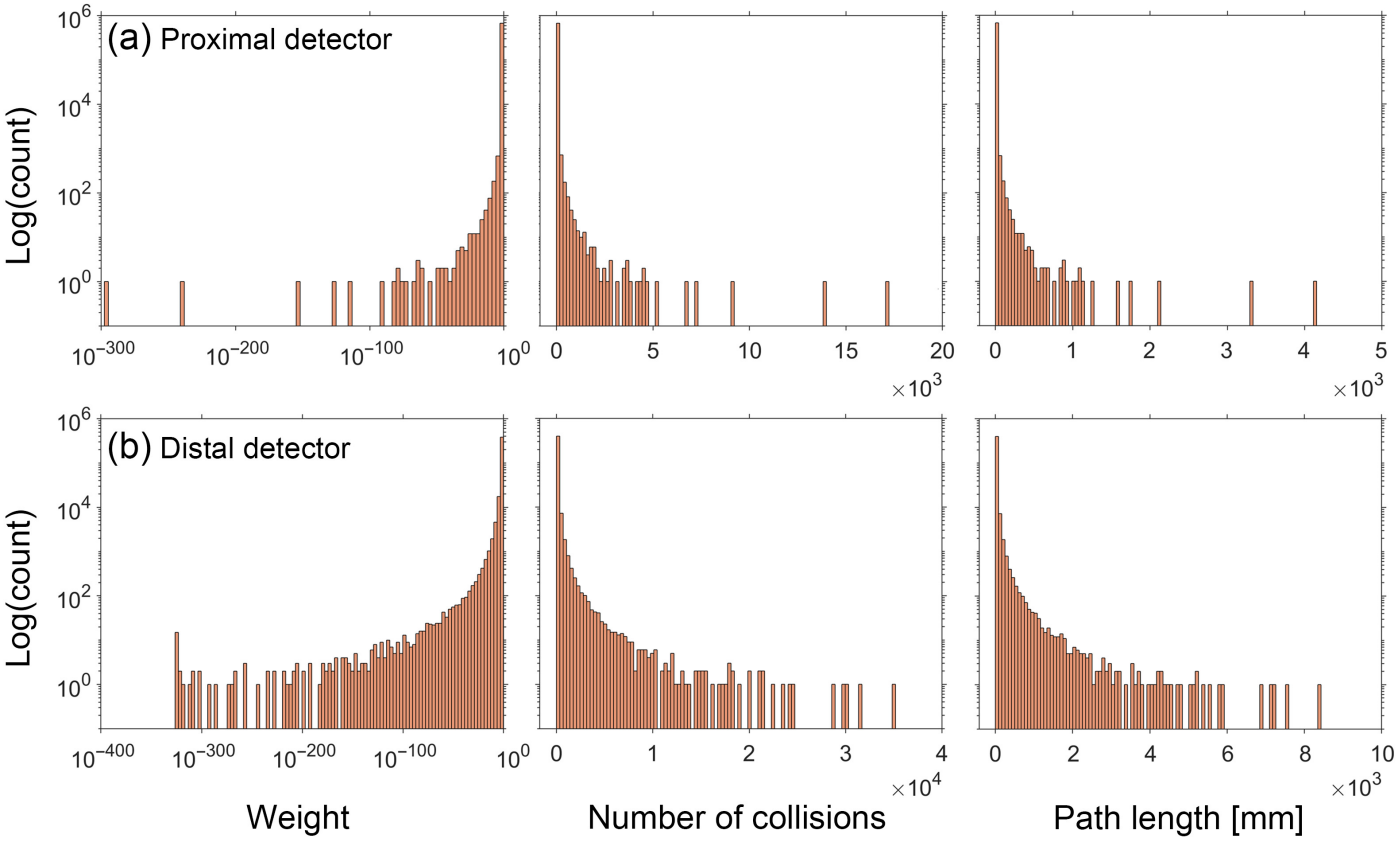
Histograms for the detected photons tallying $W_{R}$, j, and L for the reference simulation performed at $(\mu_{s}^{\prime }/\mu_{a})=5$. $N_{T}$ = 680,088 and 411,540 for the (a) proximal and (b) distal detector, respectively.

**Fig. 5:**
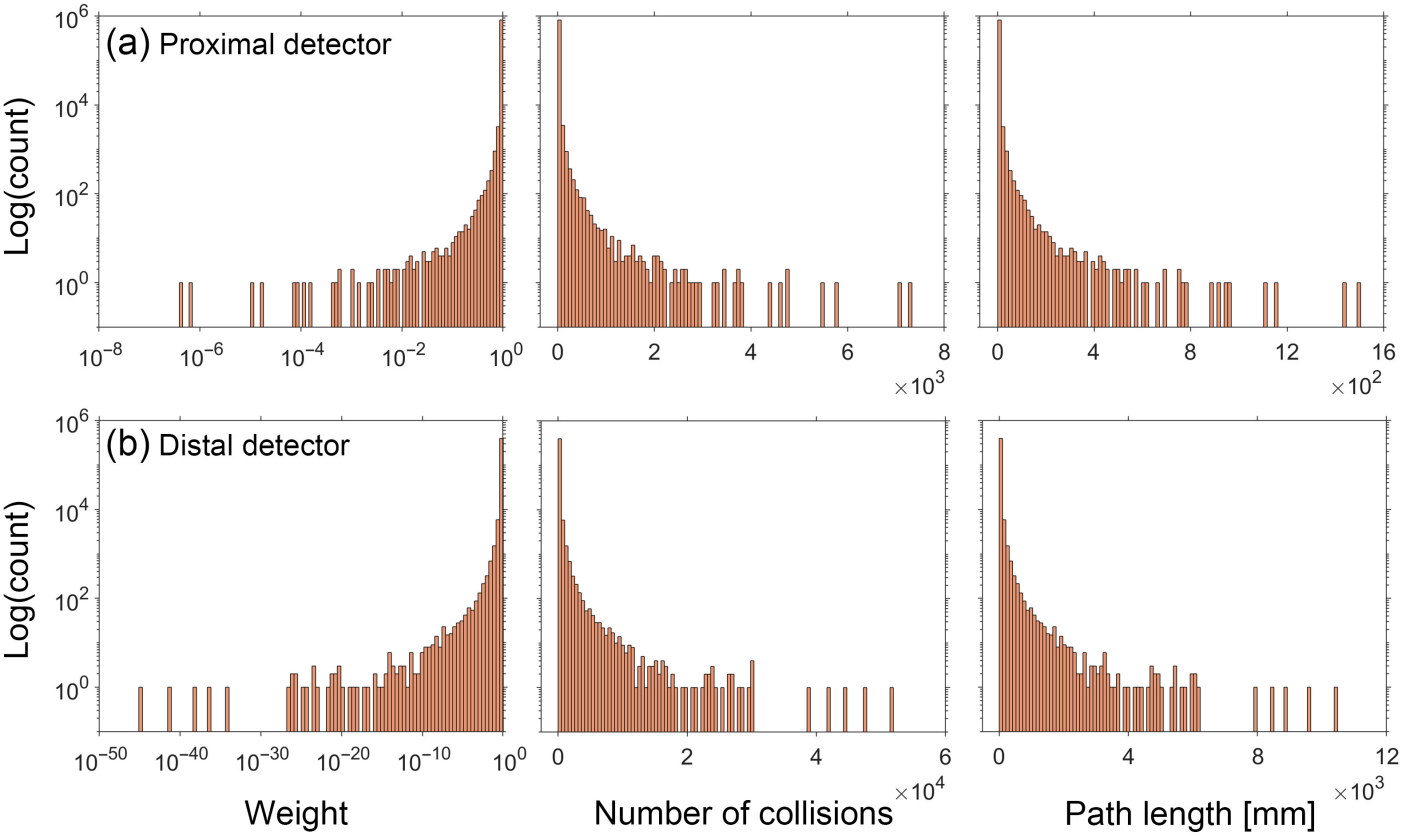
Histograms for the detected photons tallying $W_{R}$, j, and L for the reference simulation performed at $(\mu_{s}^{\prime }/\mu_{a})=100$. $N_{T}=817,515$ and 405,562 for the (a) proximal and (b) distal detector, respectively.

To gain insight into the performance characteristics of our method to estimate pMC relative error, we examined the distributions of the photon weight $W_{R}$, number of collisions j, and photon path length L for both proximal and distal detector locations. The distributions for the reference cases with $\left(\mu_{s}^{\prime }/\mu_{a}\right)$ of 5 and 100 are shown in Figs. 4 and 5, respectively. Distributions for the other three cases can be found in Section C of the Supplemental Material.

For both cases shown in Figs. 4 and 5, we observe strongly skewed distributions with long, sparsely populated tails. This feature is stronger in case of the distal detector for all reference simulations. Given that the original error propagation formula is based on a Taylor series expansion and assumes that the random variables are normally distributed, the performance of the estimation provided by Eq. (3) degrades when applied to random variables that deviate from these conditions.

To improve the accuracy of our method, we attempt to reduce the magnitude of the moments of the photon weight, path length, and collision distributions by utilizing RR^26^ in our reference simulations. RR is an unbiased method for terminating simulated photons once their weight falls below a predefined threshold with a fair game probability. Once a photon’s weight drops below a specified weight threshold during its propagation, at the next collision its weight is either amplified by a factor of 1/p with a survival probability p or terminated with probability (1−p).

**Fig. 6:**
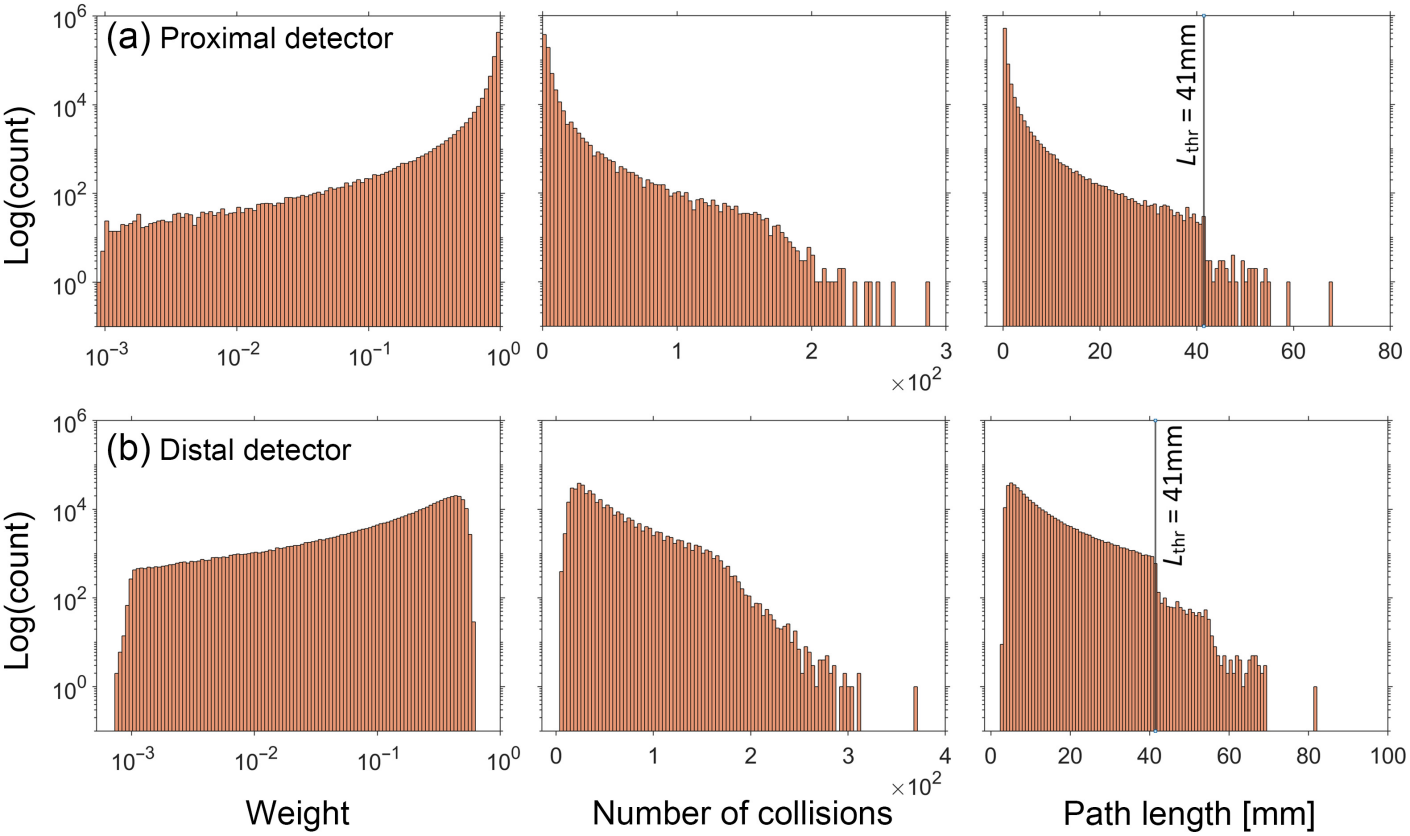
Histograms for the detected photons tallying $W_{R}$, j, and L for the reference simulation performed at $(\mu_{s}^{\prime }/\mu_{a})=5$ using RR. $N_{T}=681,805$ and 382,485 for the (a) proximal and (b) distal detector, respectively. $L_{\mathrm{thr}}$ indicates the path length corresponding to the RR weight threshold.

**Fig. 7:**
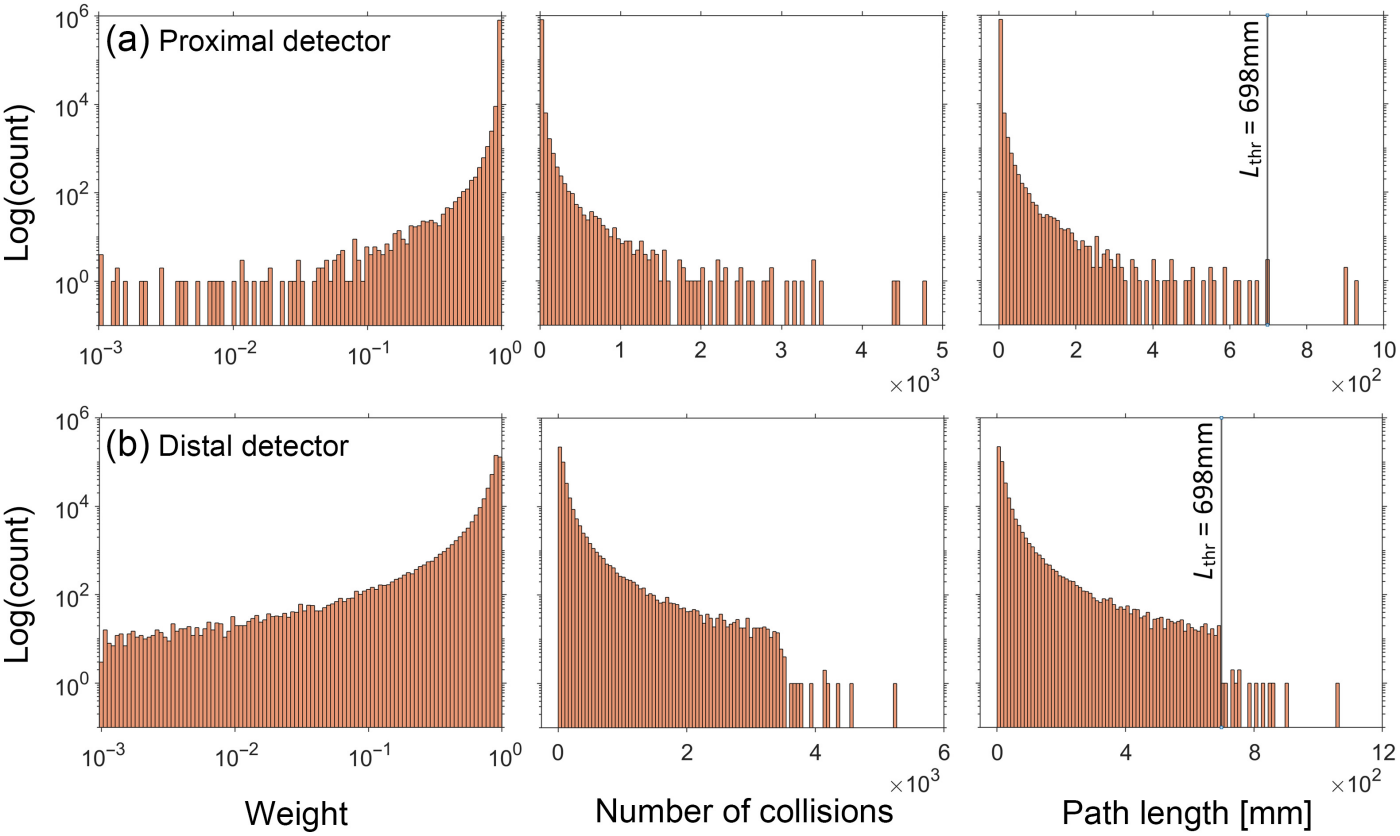
Histograms for the detected photons tallying $W_{R}$, j, and L for the reference simulation performed at $(\mu_{s}^{\prime }/\mu_{a})=100$ using RR. $N_{T}=816,995$ and 404,955 for the (a) proximal and (b) distal detector, respectively. $L_{\mathrm{thr}}$ indicates the path length corresponding to the RR weight threshold.

Using this general approach, we selected a weight threshold of $10^{-3}$ and a survival probability of 0.1. Figures 6 and 7 show the distribution of the same random variables shown in Figs. 4 and 5 after implementation of RR for the reference simulations with $\left(\mu_{s}^{\prime }/\mu_{a}\right)$ of 5 and 100, respectively. Distributions for the other three cases can be found in Section D of the Supplemental Material. Appearance of discontinuities in the j and L histograms is reflective of the tallied photon subpopulation that has undergone the RR. Comparison of the histograms utilizing the RR method with their counterparts in Figs. 4 and 5 illustrates a reduced range of values for each random variable and more compact and densely populated distributions.

We computed the various moments for the reference simulations both before and after implementation of RR and analyzed them after normalization to eliminate any dependence on the actual values. Tables 2 and 3 show the normalized characteristics of the distribution of random variables corresponding to the reference MC simulations for $\left(\mu_{s}^{\prime }/\mu_{a}\right)$ of 5 and 100, respectively, before and after applying RR.

**Table 2:** Normalized variance $\left(\sigma_{x}^{2}/{\overset{¯}{x}}^{2}\right)$, covariance $\left(\sigma_{x,y}/\sigma_{x}\sigma_{y}\right)$, and skewness $\gamma_{x}$ metrics of the logarithm of the photon weight [log ($W_{R}$)], number of photon collisions (j), and photon path length (L) for reference MC simulations performed at $(\mu_{s}^{\prime }/\mu_{a})=5$ before and after applying the RR method.

**cMC**					**cMC with RR**				
	Variable	$\sigma_{x}^{2}/{\overset{¯}{x}}^{2}$	$\sigma_{x,y}/\sigma_{x}\sigma_{y}$	$\gamma_{x}$		Variable	$\sigma_{x}^{2}/{\overset{¯}{x}}^{2}$	$\sigma_{x,y}/\sigma_{x}\sigma_{y}$	$\gamma_{x}$
**Proximal**	$\mathrm{Log}(W_{R})$	101.9	—	−194.8	**Proximal**	$\mathrm{Log}(W_{R})$	7.104	—	−8.598
	j	65.77	—	194.4		j	4.360	—	8.643
	L	101.9	—	194.8		L	7.191	—	8.810
	$\mathrm{Log}(W_{R})$, j	—	−0.999	—		$\mathrm{Log}(W_{R})$, j	—	−0.979	—
	$\mathrm{Log}(W_{R})$, L	—	−1.000	—		$\mathrm{Log}(W_{R})$, L	—	−0.999	—
	j, L	—	0.999	—		j, L	—	0.980	—
**Distal**	$\mathrm{Log}(W_{R})$	12.07	—	−31.84	**Distal**	$\mathrm{Log}(W_{R})$	0.489	—	−1.642
	j	13.68	—	40.67		j	0.536	—	1.724
	L	13.65	—	40.73		L	0.505	—	1.760
	$\mathrm{Log}(W_{R})$, j	—	−0.988	—		$\mathrm{Log}(W_{R})$, j	—	−0.976	—
	$\mathrm{Log}(W_{R})$, L	—	−0.989	—		$\mathrm{Log}(W_{R})$, L	—	−0.995	—
	j, L	—	0.999	—		j, L	—	0.980	—

**Table 3:** Normalized variance $\left(\sigma_{x}^{2}/{\overset{¯}{x}}^{2}\right)$, covariance $\left(\sigma_{x,y}/\sigma_{x}\sigma_{y}\right)$ and skewness $\gamma_{x}$ metrics of the logarithm of the photon weight [log ($W_{R}$)], number of photon collisions (j), and photon path length (L) for reference MC simulations performed at $(\mu_{s}^{\prime }/\mu_{a})=100$ before and after applying the RR method.

**cMC**					**cMC with RR**				
	Variable	$\sigma_{x}^{2}/{\overset{¯}{x}}^{2}$	$\sigma_{x,y}/\sigma_{x}\sigma_{y}$	$\gamma_{x}$		Variable	$\sigma_{x}^{2}/{\overset{¯}{x}}^{2}$	$\sigma_{x,y}/\sigma_{x}\sigma_{y}$	$\gamma_{x}$
**Proximal**	$\mathrm{Log}(W_{R})$	47.87	—	−84.50	**Proximal**	$\mathrm{Log}(W_{R})$	34.50	—	−52.49
	j	31.99	—	83.98		j	24.15	—	57.76
	L	47.87	—	84.50		L	35.90	—	57.77
	$\mathrm{Log}(W_{R})$, j	—	−0.998	—		$\mathrm{Log}(W_{R})$, j	—	−0.994	—
	$\mathrm{Log}(W_{R})$, L	—	−1.000	—		$\mathrm{Log}(W_{R})$, L	—	−0.997	—
	j, L	—	0.998	—		j, L	—	0.997	—
**Distal**	$\mathrm{Log}(W_{R})$	14.62	—	−45.00	**Distal**	$\mathrm{Log}(W_{R})$	3.614	—	−8.420
	j	14.70	—	44.94		j	3.673	—	8.515
	L	14.62	—	45.00		L	3.642	—	8.556
	$\mathrm{Log}(W_{R})$, j	—	−1.000	—		$\mathrm{Log}(W_{R})$, j	—	−0.998	—
	$\mathrm{Log}(W_{R})$, L	—	−1.000	—		$\mathrm{Log}(W_{R})$, L	—	−0.999	—
	j, L	—	1.000	—		j, L	—	0.998	—

Based on these results, the normalized variance for all random variables decreases after applying RR technique. Similarly, a reduction in normalized covariance (correlation) is observed. This makes sense since the photon reweighting accomplished by RR weakens the strict dependence between the photon weight and both path length and number of collisions. The skewness of the random variable distributions also reduced dramatically after using RR. It is also clear that the reductions are less dramatic for the reference with $(\mu_{s}^{\prime }/\mu_{a})=100$ compared with $(\mu_{s}^{\prime }/\mu_{a})=5$. This is because for $(\mu_{s}^{\prime }/\mu_{a})=100$, fewer photons undergo RR because of the larger path length is necessary for the threshold photon weight to be reached.

**Fig. 8:**
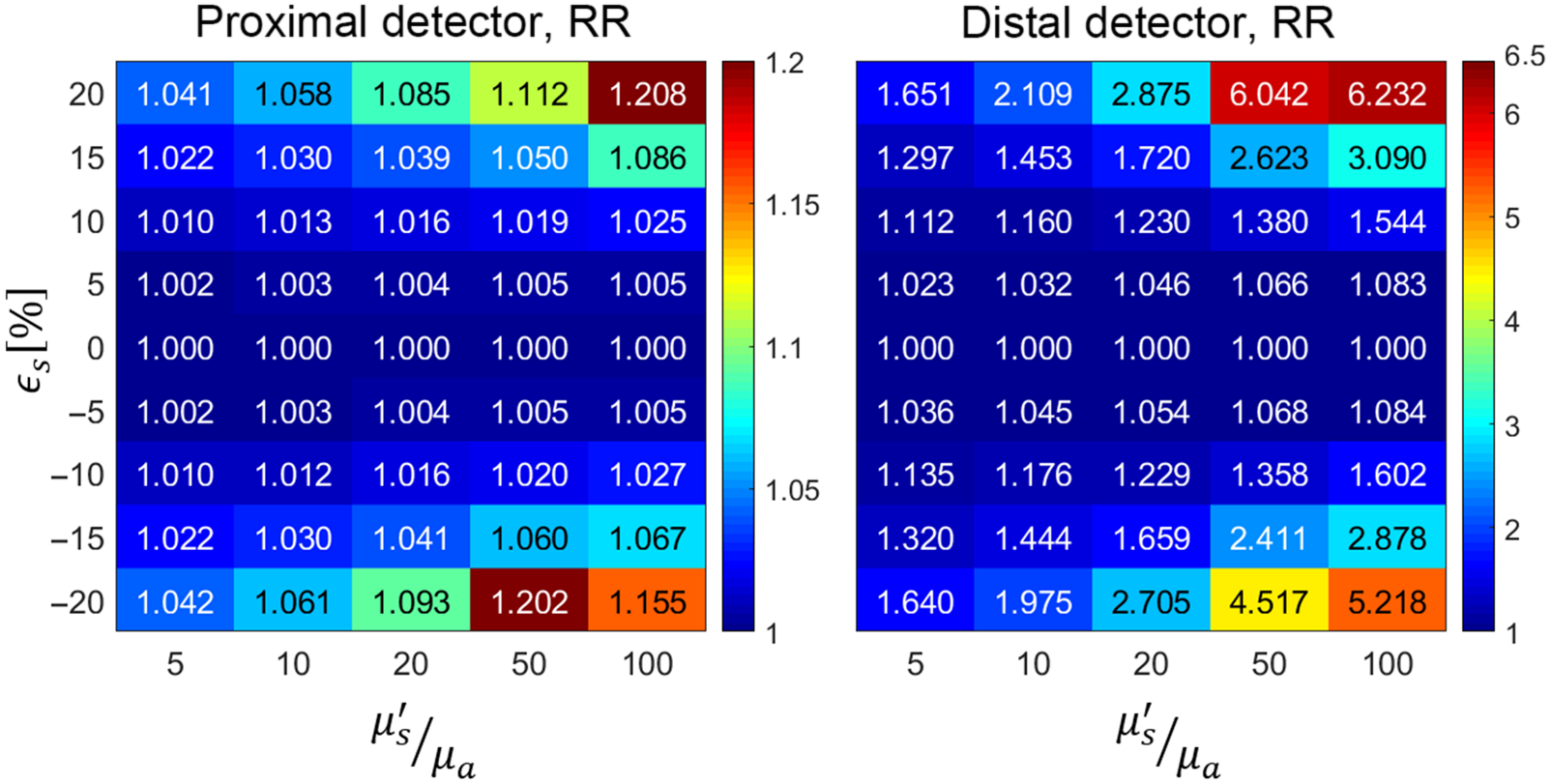
Variation of the degradation degree, ${\overset{¯}{\Delta }}_{P}$, with scattering perturbation size $\epsilon_{s}$ and optical properties $\left(\mu_{s}^{\prime }/\mu_{a}\right)$ using RR in the reference simulations.

Using the photon databases generated from these new reference simulations that utilized RR, we again performed a rigorous calculation of the pMC relative error as well as estimated the relative error utilizing only the distribution characteristics of the reference simulation data and our error propagation method. Figure 8 shows the degradation degree metric indicating how the pMC relative error varies with perturbation size using the RR reference simulations. These results are comparable to those shown in Fig. 2, which reports the same metric for the reference simulations that were performed without use of RR.

**Fig. 9:**
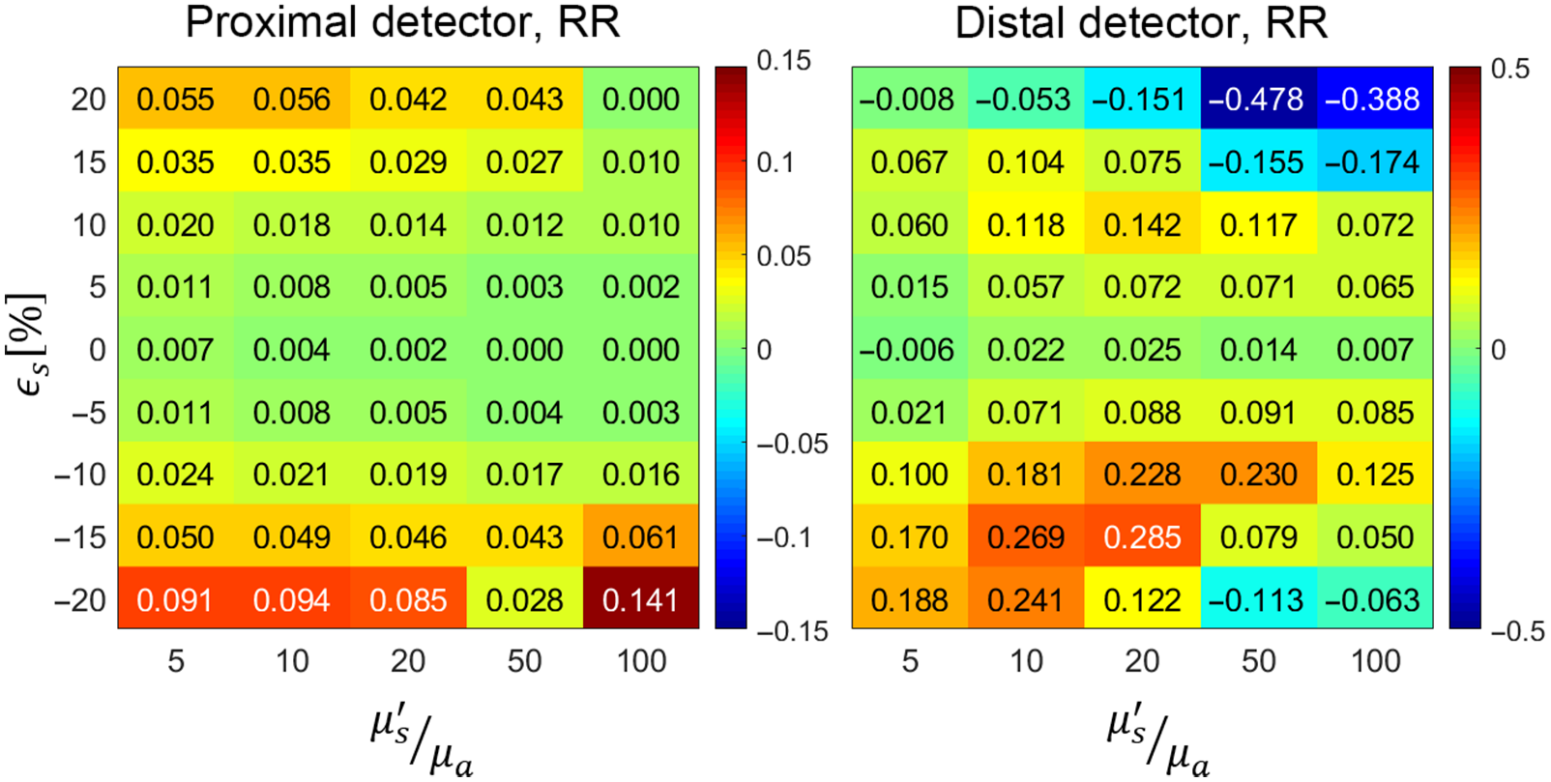
Variation of δ with scattering perturbation size $\epsilon_{s}$ and optical properties $\left(\mu_{s}^{\prime }/\mu_{a}\right)$ using RR in the reference simulations.

Figure 9 shows the relative difference δ between our estimate and the actual pMC relative error.

The utilization of RR clearly improves our ability to estimate pMC relative error using data from the reference simulation alone. For the proximal detector, the use of RR improves our pMC relative error estimates most notably for scattering perturbations beyond ±8%. With the usage of RR, we can estimate the pMC relative error within 5% of the true value for scattering perturbations in the range of [−15%,+20%]. Our predictions for the distal detector are also notably improved and the usage of RR provides relative error estimates within 20% for scattering perturbations in the range of [−8%,+15%]. For the distal detector, reference simulation with $(\mu_{s}^{\prime }/\mu_{a})=5$ seems to provide the largest range of perturbation for which our approximation method provides the highest accuracy. While the accuracy of our method is very strong overall for the proximal detector, we continue to observe poorer performance for higher values of $\left(\mu_{s}^{\prime }/\mu_{a}\right)$, which is also the case for the distal detector.

To explain the underlying reasons for this, we should first note that the use of reference simulations utilizing RR improved the overall pMC error predictions more so for the distal detector as compared with the proximal detector. Improvement in the prediction accuracy was observed to be greater for lower values of $\left(\mu_{s}^{\prime }/\mu_{a}\right)$. These characteristics are expected as photons collected at the distal detector typically have a larger path length as compared with those collected at the proximal detector. Moreover, photons propagating in the more highly absorbing medium, $(\mu_{s}^{\prime }/\mu_{a})=5$, need only travel a path length of 41 mm before RR is invoked as opposed to 698 mm for the highly scattering medium of $(\mu_{s}^{\prime }/\mu_{a})=100$. As a result, for the $(\mu_{s}^{\prime }/\mu_{a})=5$ medium, 470 and 13,550 photons underwent RR reweighting for the proximal and distal detector, respectively. By contrast for the $(\mu_{s}^{\prime }/\mu_{a})=100$ medium, only 40 and 140 of the detected photons underwent RR reweighting for the proximal and distal detector, respectively.

Taken collectively, our results suggest that reference simulations should be run using the lowest possible value of $\left(\mu_{s}^{\prime }/\mu_{a}\right)$ since the intrinsic growth of the pMC variance, as reported by the degradation degree metric ${\overset{¯}{\Delta }}_{P}$, is minimized. This result is consistent with the theoretical analysis of Ref. 20 that shows the perturbation range over which pMC estimates can be obtained, grows as the probability of scattering is decreased. Moreover, it is also reassuring to observe that our ability to estimate the pMC relative error using reference simulation data alone performs best for lower $\left(\mu_{s}^{\prime }/\mu_{a}\right)$ values. The results also show that the use of RR reduces the intrinsic variance of the reference simulations while also improving our ability to accurately estimate the pMC variance. Moreover, the threshold weight should be chosen to adequately limit excessively long path lengths that result in distributions of photon weight, path length, and collisions with extended, sparsely populated tails.

## 5 Conclusions

In conclusion, we have presented a method to estimate the relative error associated with the use of perturbation Monte Carlo estimates using distribution metrics of the reference simulation data alone. This ability reduces pMC computational cost and provides specific guidance for the selection of optical properties for the placement of reference simulations. Moreover, we have shown that the use of RR is advantageous in reducing the intrinsic relative error characteristics of reference simulations used for deriving pMC estimates as well as providing a large improvement in the perturbation range over which we can predict the relative error. Our results show conclusively that the range of scattering perturbation while minimizing the growth in the relative error of the resulting pMC estimates is best accomplished when $\left(\mu_{s}^{\prime }/\mu_{a}\right)$ is low. This result is consistent with the analysis of Ref. 20 who showed that the allowable perturbation range of pMC grows as the probability of scattering is decreased. Our results suggest that to utilize pMC for predictions over a wide range of optical properties, reference simulations should utilize CAW with optical properties corresponding to low $\left(\mu_{s}^{\prime }/\mu_{a}\right)$ values over the desired range of $\mu_{s}$ values. This is because absorption perturbations can be computed exactly when using CAW and can then be employed to compute pMC estimates for cases where $\left(\mu_{s}^{\prime }/\mu_{a}\right)$ is large. Finally, we note that the results provided in this paper represent a “worst case” for the application of pMC in that we perturbed the properties of the entire medium. However, in most applications, the perturbation will be applied to only a subdomain of the entire volume being considered. In these cases, we expect that our methodology will provide accurate results for a larger range of scattering perturbations.

## Code, Data, and Materials Availability

The MCCL open-source Monte Carlo computation engine was used to generate the results of this study and can be accessed here: https://virtualphotonics.org/software-mccl.

## References

[1] Hayakawa C. K., “Perturbation Monte Carlo methods for the solution of inverse problems,” PhD Thesis, Claremont Graduate University (2002).

[2] Zhu C. Liu Q., “Review of Monte Carlo modeling of light transport in tissues,” J. Biomed. Opt. 18(5), 050902 (2013). JBOPFO 1083-3668 10.1117/1.JBO.18.5.050902 23698318

[3] Hennessy R. J. et al., “Monte Carlo lookup table-based inverse model for extracting optical properties from tissue-simulating phantoms using diffuse reflectance spectroscopy,” J. Biomed. Opt. 18(3), 037003 (2013). JBOPFO 1083-3668 10.1117/1.JBO.18.3.037003 23455965 PMC3584151

[4] Graaff R. et al., “Condensed Monte Carlo simulations for the description of light transport,” Appl. Opt. 32(4), 426–434 (1993). APOPAI 0003-6935 10.1364/AO.32.000426 20802708

[r5] Kienle A. Patterson M. S., “Determination of the optical properties of turbid media from a single Monte Carlo simulation,” Phys. Med. Biol. 41(10), 2221–2227, (1996). 10.1088/0031-9155/41/10/026 8912392

[r6] Liu Q. Ramanujam N., “Scaling method for fast Monte Carlo simulation of diffuse reflectance spectra from multilayered turbid media,” J. Opt. Soc. Am. A 24(4), 1011–1025 (2007). JOAOD6 0740-3232 10.1364/JOSAA.24.001011 17361287

[r7] Martinelli M. et al., “Analysis of single Monte Carlo methods for prediction of reflectance from turbid media,” Opt Express 19(20), 19627–19642 (2011). OPEXFF 1094-4087 10.1364/OE.19.019627 21996904 PMC3347703

[8] Palmer G. M. Ramanujam N., “Monte Carlo-based inverse model for calculating tissue optical properties. Part I: Theory and validation on synthetic phantoms,” Appl. Opt. 45(5), 1062–1071 (2006). APOPAI 0003-6935 10.1364/AO.45.001062 16512550

[9] Hayakawa C. K. et al., “Perturbation Monte Carlo methods to solve inverse photon migration problems in heterogeneous tissues,” Opt. Lett. 26(17), 1335–1337, (2001). OPLEDP 0146-9592 10.1364/OL.26.001335 18049600

[10] Alerstam E. Svensson T. Andersson-Engels S., “Parallel computing with graphics processing units for high-speed Monte Carlo simulation of photon migration,” J. Biomed. Opt. 13(6), 060504 (2008). JBOPFO 1083-3668 10.1117/1.3041496 19123645

[r11] Colasanti A. et al., “Multiple processor version of a Monte Carlo code for photon transport in turbid media,” Comput. Phys. Commun. 132(1-2), 84–93 (2000). CPHCBZ 0010-4655 10.1016/S0010-4655(00)00138-7

[12] Fang Q. Yan S., “MCX Cloud: a modern, scalable, high-performance and in-browser Monte Carlo simulation platform with cloud computing,” J. Biomed. Opt. 27(8), 083008 (2022). JBOPFO 1083-3668 10.1117/1.JBO.27.8.083008 34989198 PMC8728956

[13] Lima I. T. Kalra A. Sherif S. S., “Improved importance sampling for Monte Carlo simulation of time-domain optical coherence tomography,” Biomed. Opt. Express 2(5), 1069–1081 (2011). BOEICL 2156-7085 10.1364/BOE.2.001069 21559120 PMC3087565

[14] Seo I. et al. “Perturbation and differential Monte Carlo methods for measurement of optical properties in a layered epithelial tissue model,” J. Biomed. Opt. 12(1), 014030 (2007). JBOPFO 1083-3668 10.1117/1.2697735 17343505

[r15] Kumar Y. P. Vasu R. M., “Reconstruction of optical properties of low-scattering tissue using derivative estimated through perturbation Monte-Carlo method,” J. Biomed. Opt. 9(5), 1002–1012 (2004). JBOPFO 1083-3668 10.1117/1.1778733 15447022

[16] Leino A. A. et al., “Perturbation Monte Carlo method for quantitative photoacoustic tomography,” IEEE Trans. Med. Imaging 39(10), 2985–2995 (2020). ITMID4 0278-0062 10.1109/TMI.2020.2983129 32217473

[17] Sassaroli A., “Fast perturbation Monte Carlo method for photon migration in heterogeneous turbid media,” Opt. Lett. 36(11), 2095–2097 (2011). OPLEDP 0146-9592 10.1364/OL.36.002095 21633460 PMC3267237

[r18] Nguyen J. et al., “Development of perturbation Monte Carlo methods for polarized light transport in a discrete particle scattering model,” Biomed. Opt. Express 7(5):2051–2066 (2016). BOEICL 2156-7085 10.1364/BOE.7.002051 27231642 PMC4871102

[19] Nguyen J. et al., “Perturbation Monte Carlo methods for tissue structure alterations,” Biomed. Opt. Express 4(10), 1946–1963 (2013). BOEICL 2156-7085 10.1364/BOE.4.001946 24156056 PMC3799658

[20] Rief H., “Generalized Monte Carlo perturbation algorithms for correlated sampling and a second-order Taylor series approach,” Ann. Nucl. Energy 11(9):455–476 (1984). ANENDJ 0306-4549 10.1016/0306-4549(84)90064-1

[21] Yamamoto T. Sakamoto H., “Frequency domain optical tomography using a Monte Carlo perturbation method,” Opt. Commun. 364:165–176 (2016). OPCOB8 0030-4018 10.1016/j.optcom.2015.11.055

[22] Yao R. Intes X. Fang Q., “Direct approach to compute Jacobians for diffuse optical tomography using perturbation Monte Carlo-based photon “replay”,” Biomed. Opt. Express 9(10), 4588–4603 (2018). BOEICL 2156-7085 10.1364/BOE.9.004588 30319888 PMC6179418

[23] Hayakawa C. K. Spanier J. Venugopalan V., “Comparative analysis of discrete and continuous absorption weighting estimators used in Monte Carlo simulations of radiative transport in turbid media,” J. Opt. Soc. Am. A 31(2), 301–311 (2014). JOAOD6 0740-3232 10.1364/JOSAA.31.000301 PMC4106716 24562029

[24] Ku H. H., “Notes on the use of propagation of error formulas,” J. Res. Nat. Bur. Stand. 70(4), 263–273 (1966). 10.6028/jres.070C.025

[25] Anderson T. V. Mattson C. A., “Propagating skewness and kurtosis through engineering models for low-cost, meaningful, nondeterministic design,” ASME J. Mech. Des. 134(10), 100911 (2012). 10.1115/1.4007389

[26] Hayakawa C. K. et al., “MCCL: an open-source software application for Monte Carlo simulations of radiative transport,” J. Biomed. Opt. 27(8), 083005 (2022). JBOPFO 1083-3668 10.1117/1.JBO.27.8.083005 35415991 PMC9005200

[27] Parsanasab M. et al., “Uncertainty analysis in perturbation Monte Carlo simulations of radiative transport,” Proc. SPIE 12376, 21–25 (2023). PSISDG 0277-786X 10.1117/12.2650969 PMC10245552 37293394

