# Supplementary material for: Analysis of relative error in perturbation Monte Carlo simulations of radiative transport
Source: J Biomed Opt. 2023 Jun 7;28(6):065001. doi: 10.1117/1.JBO.28.6.065001 (PMC10245552; doi:10.1117/1.JBO.28.6.065001)
Supplement: Supplementary file 1 [file JBO_028_065001_SD001.pdf]

Supplemental Materials
Analysis of relative error in perturbation Monte Carlo simulations of radiative transport
by M Parsanasab, CK Hayakawa, J Spanier, Y Shen, and V Venugopalan

Section A: Derivation of Equation for The Variance of Perturbed Weight

Our objective is to estimate the variance σ^2 of a population of perturbed weights W_P based on the population characteristics of the photon weights obtained from a reference simulation W_R. We begin with the basic equation defining the variance of a population of perturbed weights W_P:

$$\sigma_{W_\mathrm{P}}^2 = \frac{1}{N}\sum_{i=1}^{N}\left(W_{\mathrm{P},i} - \bar{W}_\mathrm{P}\right)^2 \quad \text{(A1)}$$

For a perturbation Monte Carlo simulation each perturbed photon weight is computed from a corresponding reference photon weight as follows:

$$W_{\mathrm{P},i} = W_{\mathrm{R},i}\left(\frac{\mu_{s,\mathrm{P}}}{\mu_{s,\mathrm{R}}}\right)^{j_i} \exp\left[-\left(\mu_{t,\mathrm{P}} - \mu_{t,\mathrm{R}}\right)L_i\right] \quad \text{(A2)}$$

In Eq. (A2), μ_s and μ_t are the scattering and total interaction coefficients, respectively, j is the number of collisions the photon experiences in the medium, and L is the photon path length. The subscripts P and R refer to the perturbed and reference cases, respectively. Eq. (A2) applies only to those photons tallied at the detector under consideration. Assuming for the moment that the variables $W_{\mathrm{R},i}$, j_i, and L_i are not totally independent and not governed by a normal distribution, the variance of the distribution of perturbed weights can be estimated by:

$$\sigma_{W_\mathrm{P}}^2 \approx \left(\frac{\partial W_\mathrm{P}}{\partial W_\mathrm{R}}\right)^2 \sigma_{W_\mathrm{R}}^2 + \left(\frac{\partial W_\mathrm{P}}{\partial j}\right)^2 \sigma_j^2 + \left(\frac{\partial W_\mathrm{P}}{\partial L}\right)^2 \sigma_L^2$$

$$+2\left(\frac{\partial W_\mathrm{P}}{\partial W_\mathrm{R}}\right)\left(\frac{\partial W_\mathrm{P}}{\partial j}\right)\sigma_{W_\mathrm{R},j} + 2\left(\frac{\partial W_\mathrm{P}}{\partial W_\mathrm{R}}\right)\left(\frac{\partial W_\mathrm{P}}{\partial L}\right)\sigma_{W_\mathrm{R},L} + 2\left(\frac{\partial W_\mathrm{P}}{\partial j}\right)\left(\frac{\partial W_\mathrm{P}}{\partial L}\right)\sigma_{j,L} \quad \text{(A3)}$$

$$+\left(\frac{\partial W_\mathrm{P}}{\partial W_\mathrm{R}}\right)\left(\frac{\partial^2 W_\mathrm{P}}{\partial W_\mathrm{R}^2}\right)\gamma_{W_\mathrm{R}}\sigma_{W_\mathrm{R}}^3 + \left(\frac{\partial W_\mathrm{P}}{\partial j}\right)\left(\frac{\partial^2 W_\mathrm{P}}{\partial j^2}\right)\gamma_j\sigma_j^3 + \left(\frac{\partial W_\mathrm{P}}{\partial L}\right)\left(\frac{\partial^2 W_\mathrm{P}}{\partial L^2}\right)\gamma_L\sigma_L^3$$

When applying Eq. (A3) to a population of reference weights, we must be aware that only a subset of the N photons that are simulated are tallied at the detector (N_T) and have their weights modified according to the relationship in Eq. (A2) while the remaining photons (N_U) go untallied at the detector.

We thus reformulate Eq (A1) that defines the variance of the perturbed estimate $\sigma_{W_\mathrm{P}}^2$, in a manner that separates the contributions of the tallied and untallied photons to the variance of the perturbed photon weight. We thus rewrite Eq. (A1) as follows:

$$\sigma_{W_\mathrm{P}}^2 = \frac{1}{N}\left[\sum_{N_\mathrm{T}}\left(W_{\mathrm{P},i} - \bar{W}_\mathrm{P}\right)^2 + \sum_{N_\mathrm{U}}\left(W_{\mathrm{P},i} - \bar{W}_\mathrm{P}\right)^2\right] \tag{A4}$$

If we consider the photons that are untallied, we recognize that $W_\mathrm{P} = W_\mathrm{R} = 0$. Thus, the contribution of the untallied photons to the variance is simply equal to $\frac{N_\mathrm{U}}{N} \times \bar{W}_\mathrm{P}^2$. We are then left to determine the first term within the square brackets, i.e., the contribution of the tallied photons to the variance. Let us rewrite this term in a manner as to explicitly introduce the mean weight of only those photons that were tallied:

$$\begin{aligned}\sum_{N_\mathrm{T}}\left(W_{\mathrm{P},i} - \bar{W}_\mathrm{P}\right)^2 &= \sum_{N_\mathrm{T}}\left(W_{\mathrm{P},i} - \bar{\boldsymbol{W}}_{\mathbf{P,T}} + \bar{\boldsymbol{W}}_{\mathbf{P,T}} - \bar{W}_\mathrm{P}\right)^2 \\ &= \sum_{N_T}\left[\left(W_{\mathrm{P},i} - \bar{W}_\mathrm{P,T}\right) - \left(\bar{W}_\mathrm{P} - \bar{W}_\mathrm{P,T}\right)\right]^2\end{aligned} \tag{A5}$$

where $\bar{W}_\mathrm{P,T} = \frac{1}{N_\mathrm{T}}\sum_{N_\mathrm{T}} W_{\mathrm{P},i}$. We note that the second term within the parenthesis on the right-hand side of Eq. (A5) is simply the difference between the mean of the entire population of simulated photons and the mean of the sub-population of tallied photons. We will term this difference $\bar{\Phi}$. Making this variable substitution and expanding the square we get:

$$\begin{aligned}\sum_{N_\mathrm{T}}\left(W_{\mathrm{P},i} - \bar{W}_\mathrm{P}\right)^2 &= \sum_{N_\mathrm{T}}\left[\left(W_{\mathrm{P},i} - \bar{W}_\mathrm{P,T}\right)^2 + \bar{\Phi}^2 - 2\bar{\Phi}\left(W_{\mathrm{P},i} - \bar{W}_\mathrm{P,T}\right)\right] \\ &= \sum_{N_\mathrm{T}}\left(W_{\mathrm{P},i} - \bar{W}_\mathrm{P,T}\right)^2 + \sum_{N_\mathrm{T}}\bar{\Phi}^2 - 2\bar{\Phi}\sum_{N_\mathrm{T}}\left(W_{\mathrm{P},i} - \bar{W}_\mathrm{P,T}\right)\end{aligned} \tag{A6}$$

The first term on the right-hand side is simply N_T multiplied by the variance of the population of tallied perturbed photon weights while the last term in this expansion is identically zero since the definition of the mean of a population requires $\sum_{N_\mathrm{T}}\left(W_{\mathrm{P},i} - \bar{W}_\mathrm{P,T}\right) = 0$. Therefore, we can arrive at the final expression for the contribution of the tallied photons to the variance of the entire population of perturbed photon weights as follows:

$$\sum_{N_\mathrm{T}}\left(W_{\mathrm{P},i} - \bar{W}_\mathrm{P}\right)^2 = N_\mathrm{T}\left(\sigma_{W_\mathrm{P,T}}^2 + \bar{\Phi}^2\right) \tag{A7}$$

Substituting this expression into Eq. (A4) gives us our final expression for the variance of the entire population of perturbed photon weights as:

$$\sigma_{W_\mathrm{P}}^2 = \frac{1}{N}\left[N_\mathrm{T}\left(\sigma_{W_\mathrm{P,T}}^2 + \bar{\Phi}^2\right) + N_\mathrm{U}\bar{W}_\mathrm{P}^2\right], \tag{A8}$$

where the variance of the weights of sub-population of tallied photons $\sigma^2_{W_{\mathrm{P,T}}} = \frac{1}{N_{\mathrm{T}}} \sum_{N_{\mathrm{T}}} \left(W_{\mathrm{P},i} - \bar{W}_{\mathrm{P}}\right)^2$. Then we can adopt Eq. (A3) to approximate $\sigma^2_{W_{\mathrm{P,T}}}$ in Eq. (A8).

With power and exponential terms in the perturbed weight equation, any small change grows exceedingly fast, as seen in Eq. (A2). As a result, the natural log of the equation is used as the original equation to use the error propagation algorithm to regulate this expansion:

$$\begin{aligned}\ln\left(W_{\mathrm{P},i}\right) &= \ln\left(W_{\mathrm{R},i}\right) + j_i \times \ln\left(\frac{\mu_{s,\mathrm{P}}}{\mu_{s,\mathrm{R}}}\right) - \left(\mu_{t,\mathrm{P}} - \mu_{t,\mathrm{R}}\right)L_i \\ &= \ln\left(W_{\mathrm{R},i}\right) + j_i \ln(S) - \epsilon_t L_i\end{aligned} \qquad \text{(A9)}$$

For simplicity we put $S = \frac{\mu_{s,\mathrm{P}}}{\mu_{s,\mathrm{R}}}$ and $\epsilon_t = \mu_{t,\mathrm{P}} - \mu_{t,\mathrm{R}}$. The form of each first and second order partial derivative within Eq. (A3) then becomes:

$$\begin{aligned}&\frac{\partial \ln(W_{\mathrm{P}})}{\partial W_{\mathrm{R}}} = \frac{1}{\bar{W}_{\mathrm{R}}}; \qquad \frac{\partial \ln(W_{\mathrm{P}})}{\partial j} = \ln(S); \qquad \frac{\partial \ln(W_{\mathrm{P}})}{\partial L} = -\epsilon_t; \\ &\frac{\partial^2 \ln(W_{\mathrm{P}})}{\partial W_{\mathrm{R}}^2} = -\frac{1}{\bar{W}_{\mathrm{R}}^2}; \qquad \frac{\partial^2 \ln(W_{\mathrm{P}})}{\partial j^2} = 0; \qquad \frac{\partial^2 \ln(W_{\mathrm{P}})}{\partial L^2} = 0;\end{aligned} \qquad \text{(A10)}$$

Utilizing the expressions of Eq. (A10) we can approximate the variance of the sub-population of tallied perturbed photon weights as follows:

$$\begin{aligned}\sigma^2_{\ln\,(W_{\mathrm{P,T}})} \approx &\left(\frac{1}{\bar{W}_{\mathrm{R,T}}}\right)^2 \sigma^2_{W_{\mathrm{R,T}}} + [\ln(S)]^2 \sigma_j^2 + (-\epsilon_t)^2 \sigma_L^2 \\ &+2\left(\frac{1}{\bar{W}_{\mathrm{R,T}}}\right)[\ln(S)]\sigma_{W_{\mathrm{R,T}},j} + 2\left(\frac{1}{\bar{W}_{\mathrm{R,T}}}\right)(-\epsilon_t)\sigma_{W_{\mathrm{R,T}},L} + 2[\ln(S)](-\epsilon_t)\sigma_{j,L} \\ &+\left(\frac{1}{\bar{W}_{\mathrm{R,T}}}\right)\left(-\frac{1}{\bar{W}^2_{\mathrm{R,T}}}\right)\gamma_{W_{\mathrm{R,T}}}\sigma^3_{W_{\mathrm{R,T}}}\end{aligned} \qquad \text{(A11)}$$

In these equations $\sigma^2_{W_{\mathrm{R,T}}}$, σ_j^2, σ_L^2, $\sigma_{W_{\mathrm{R,T}},j}$, $\sigma_{W_{\mathrm{R,T}},L}$, $\sigma_{j,L}$ and $\gamma_{W_{\mathrm{R,D}}}$are the variance of tallied photons W_{R}, j and L distributions, the covariance between pairs of random variables and the skewness of the W_{R} distribution and they are calculated using equations below:

$$\begin{aligned}&\sigma^2_{W_{\mathrm{R,T}}} = \frac{1}{N_{\mathrm{T}}}\sum_{i=1}^{N_{\mathrm{T}}}\left(W_{\mathrm{R,T},i} - \bar{W}_{\mathrm{R,T}}\right)^2; \quad \sigma_j^2 = \frac{1}{N_{\mathrm{T}}}\sum_{i=1}^{N_{\mathrm{T}}}(j_i - \bar{j})^2; \quad \sigma_L^2 = \frac{1}{N_{\mathrm{T}}}\sum_{i=1}^{N_{\mathrm{T}}}(L_i - \bar{L})^2 \\ &\sigma_{W_{\mathrm{R,T}},j} = \frac{1}{N_{\mathrm{T}}}\sum_{i=1}^{N_{\mathrm{T}}}\left(W_{\mathrm{R,T},i} - \bar{W}_{\mathrm{R,T}}\right)(j_i - \bar{j}); \quad \sigma_{W_{\mathrm{R,T}},L} = \frac{1}{N_{\mathrm{T}}}\sum_{i=1}^{N_{\mathrm{T}}}\left(W_{\mathrm{R,T},i} - \bar{W}_{\mathrm{R,T}}\right)(L_i - \bar{L})\end{aligned} \qquad \text{(A12)}$$

$$\sigma_{j,L} = \frac{1}{N_\mathrm{T}} \sum_{i=1}^{N_\mathrm{T}} (j_i - \bar{j})(L_i - \bar{L})\,; \qquad \gamma_{W_\mathrm{R,T}} = \frac{1}{N_\mathrm{T}\sigma_{W_\mathrm{R,T}}^3} \sum_{i=1}^{N_\mathrm{T}} \left(W_{\mathrm{R,T},i} - \bar{W}_\mathrm{R,T}\right)^3$$

The last step is to convert $\sigma_{\ln\,(W_\mathrm{P,T})}^2$ to $\sigma_{W_\mathrm{P,T}}^2$ so that we can substitute it in Eq. (A8). Considering $W_\mathrm{P,T}$ as x and $\ln\,(W_\mathrm{P,T})$ as y, we can relate these two terms using a second approximation, $\sigma_y^2 \approx \left(\frac{\partial y}{\partial x}\right)^2 \sigma_x^2$:

$$\sigma_{\ln\,(W_\mathrm{P,T})}^2 \approx \left(\frac{1}{\bar{W}_\mathrm{P,T}}\right)^2 \sigma_{W_\mathrm{P,T}}^2 \rightarrow\ \sigma_{W_\mathrm{P,T}}^2 \approx \left(\bar{W}_\mathrm{P,T}\right)^2 \sigma_{\ln\,(W_\mathrm{P,T})}^2 \qquad \text{(A13)}$$

Since perturbation method is based on very small changes in optical properties, and we want our estimations to utilize information from the reference simulation only, we can substitute $\bar{W}_\mathrm{P,T}$ with $\bar{W}_\mathrm{R,T}$. In this case, the above equation becomes:

$$\sigma_{W_\mathrm{P,T}}^2 \approx \left(\bar{W}_\mathrm{R,T}\right)^2 \sigma_{\ln\,(W_\mathrm{P,T})}^2 \qquad \text{(A14)}$$

Section B: Perturbed Reflectance Values for All Cases at Both Proximal and Distal Detectors

Table B1. Perturbed reflectance (×10²) and standard deviation (×10²) results at the proximal detector computed from a cMC simulation *without* using Russian Roulette.

ϵ_s[%] \ μ_s'/μ_a	**5**	**10**	**20**	**50**	**100**
**20**	3.711±0.005	4.260±0.005	4.587±0.005	4.826±0.006	4.924±0.006
**15**	3.547±0.004	4.072±0.005	4.385±0.005	4.614±0.005	4.705±0.005
**10**	3.384±0.004	3.883±0.004	4.183±0.005	4.401±0.005	4.486±0.005
**5**	3.221±0.004	3.696±0.004	3.981±0.004	4.189±0.005	4.269±0.005
**0**	3.059±0.004	3.510±0.004	3.780±0.004	3.978±0.004	4.053±0.004
**-5**	2.898±0.003	3.324±0.004	3.580±0.004	3.767±0.004	3.838±0.004
**-10**	2.737±0.003	3.139±0.004	3.381±0.004	3.557±0.004	3.623±0.004
**-15**	2.578±0.003	2.955±0.003	3.183±0.004	3.349±0.004	3.410±0.004
**-20**	2.419±0.003	2.772±0.003	2.985±0.004	3.142±0.004	3.197±0.004

Table B2. Perturbed reflectance (×10²) and standard deviation (×10²) results at the proximal computed from a cMC simulation using Russian Roulette.

ϵ_s[%] \ μ_s'/μ_a	**5**	**10**	**20**	**50**	**100**
**20**	3.732±0.005	4.255±0.005	4.584±0.005	4.826±0.006	4.914±0.006
**15**	3.566±0.004	4.065±0.005	4.380±0.005	4.613±0.005	4.697±0.005
**10**	3.401±0.004	3.877±0.004	4.176±0.005	4.400±0.005	4.481±0.005
**5**	3.236±0.004	3.689±0.004	3.974±0.004	4.188±0.005	4.265±0.005
**0**	3.073±0.004	3.502±0.004	3.773±0.004	3.976±0.004	4.050±0.004
**-5**	2.910±0.003	3.316±0.004	3.572±0.004	3.766±0.004	3.836±0.004
**-10**	2.748±0.003	3.131±0.004	3.373±0.004	3.556±0.004	3.623±0.004
**-15**	2.587±0.003	2.947±0.003	3.175±0.004	3.347±0.004	3.409±0.004
**-20**	2.427±0.003	2.764±0.003	2.978±0.004	3.139±0.004	3.197±0.004

Table B3. Perturbed reflectance (×10^2) and standard deviation (×10^2) results at the distal detector computed from a cMC simulation *without* using Russian Roulette.

μ_s'/μ_a \ ϵ_s[%]	**5**	**10**	**20**	**50**	**100**
**20**	0.429±0.001	0.738±0.003	1.065±0.006	1.421±0.010	1.613±0.023
**15**	0.440±0.001	0.755±0.002	1.090±0.003	1.459±0.005	1.654±0.010
**10**	0.450±0.001	0.771±0.002	1.113±0.002	1.490±0.003	1.687±0.004
**5**	0.4589±0.0009	0.787±0.001	1.135±0.002	1.517±0.003	1.713±0.003
**0**	0.4670±0.0009	0.801±0.001	1.155±0.002	1.539±0.002	1.736±0.003
**-5**	0.4737±0.0009	0.813±0.001	1.174±0.002	1.559±0.003	1.756±0.003
**-10**	0.479±0.001	0.824±0.002	1.190±0.002	1.576±0.003	1.771±0.004
**-15**	0.482±0.001	0.832±0.002	1.202±0.003	1.587±0.005	1.778±0.008
**-20**	0.483±0.002	0.836±0.003	1.210±0.005	1.587±0.010	1.765±0.013

Table B4. Perturbed reflectance (×10^2) and standard deviation (×10^2) results at the distal detector computed from a cMC simulation using Russian Roulette.

μ_s'/μ_a \ ϵ_s[%]	**5**	**10**	**20**	**50**	**100**
**20**	0.430±0.001	0.732±0.003	1.069±0.005	1.450±0.014	1.616±0.016
**15**	0.441±0.001	0.751±0.002	1.092±0.003	1.476±0.006	1.658±0.008
**10**	0.451±0.001	0.769±0.002	1.113±0.002	1.500±0.003	1.690±0.004
**5**	0.4597±0.0009	0.785±0.001	1.133±0.002	1.522±0.003	1.716±0.003
**0**	0.4677±0.0009	0.800±0.001	1.152±0.002	1.543±0.002	1.737±0.003
**-5**	0.4743±0.0009	0.814±0.001	1.169±0.002	1.562±0.003	1.755±0.003
**-10**	0.480±0.001	0.825±0.002	1.183±0.002	1.577±0.003	1.772±0.004
**-15**	0.483±0.001	0.835±0.002	1.194±0.003	1.590±0.006	1.779±0.008
**-20**	0.484±0.001	0.841±0.003	1.201±0.005	1.595±0.011	1.769±0.015

Section C: Histograms of Photon Weight (W_R), Number of Collision (j) and Path Length (L) from cMC Reference Simulations for $\left({\mu_s'}/{\mu_a}\right)$ = 10, 20, and 50

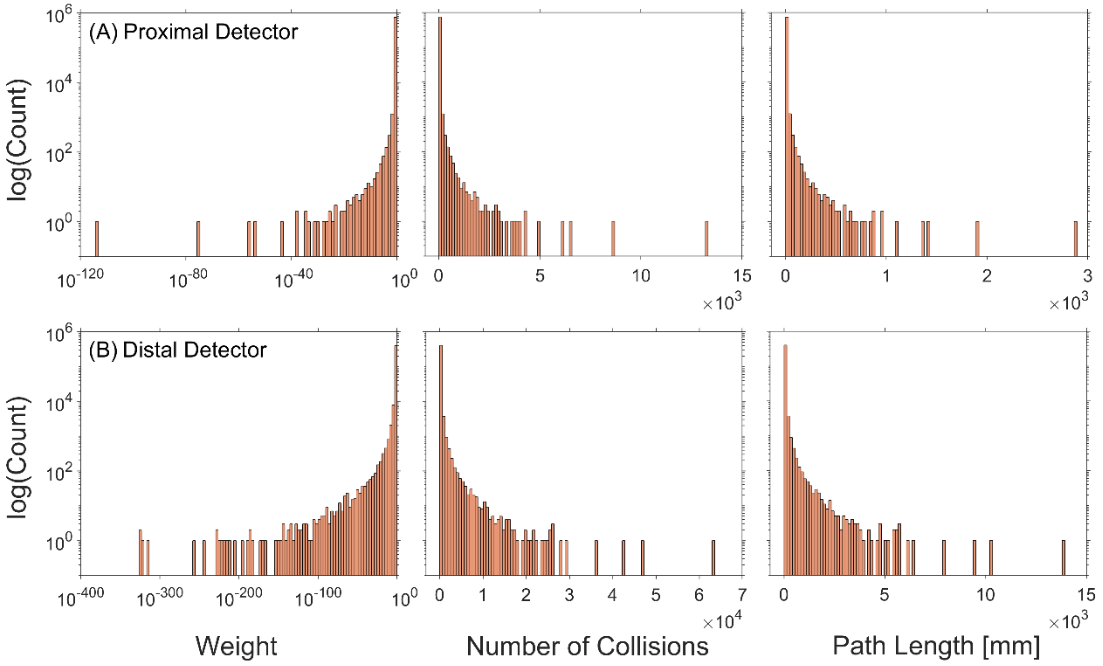

Figure C1. Histograms of W_R, j and L [mm] for tallied photons in the reference simulation performed at $\left({\mu_s'}/{\mu_a}\right)$ = 10. N_T = 747,836 and = 411,388 for the proximal and distal detector, respectively.

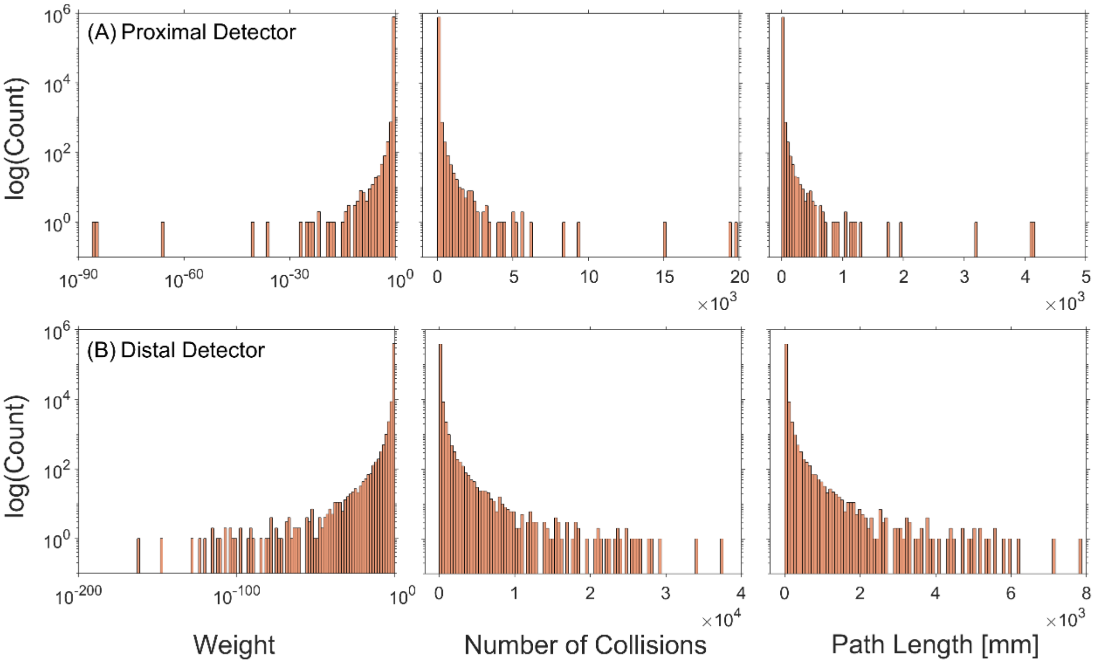

Figure C2. Histograms of W_R, j and L [mm] for tallied photons in the reference simulation performed at $\left({\mu_s'}/{\mu_a}\right)$ = 20. N_T = 783,740 and = 408,618 for the proximal and distal detector, respectively.

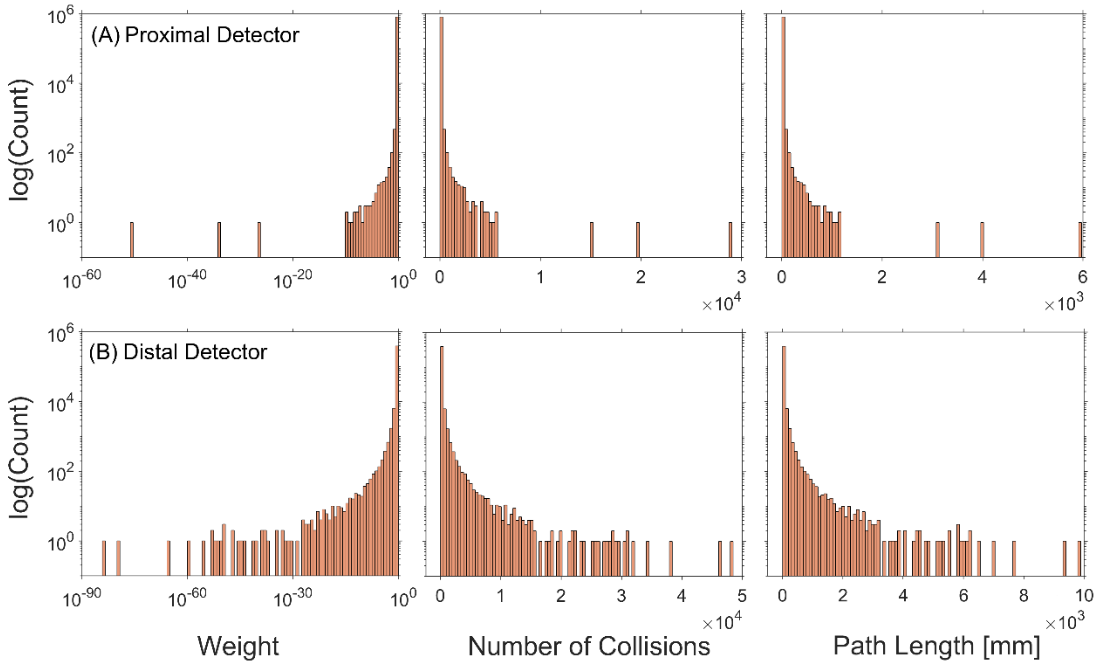

Figure C3. Histograms of W_R, j and L [mm] for tallied photons in the reference simulation performed at $\left(\mu_s'/\mu_a\right) = 50$. N_T = 808,448 and = 406,544 for the proximal and distal detector, respectively.

Section D: Histograms of Photon Weight W_R, Number of Collisions *j*, and Path Length (*L*) from cMC Reference Simulations with Russian Roulette for $\left(\frac{\mu_s'}{\mu_a}\right)$ = 10, 20, and 50

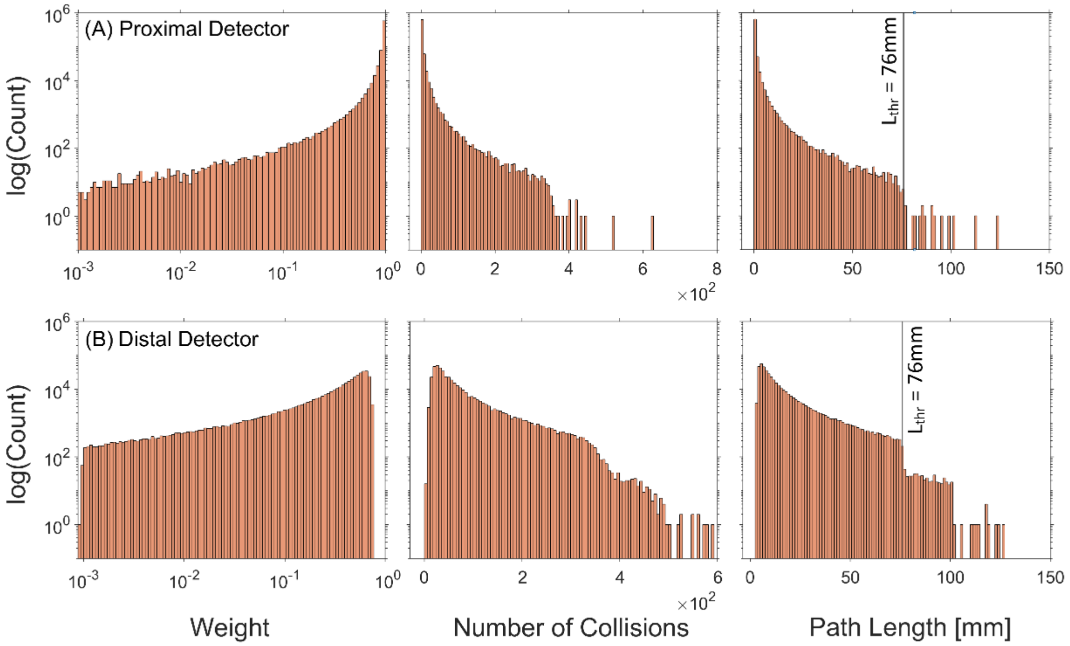

Figure D1. Histograms of W_R, *j* and *L* [mm] for tallied photons in the reference simulation performed at $\left(\mu_s'/\mu_a\right)$ = 10 using Russian Roulette. N_T = 745,965 and 397,602 for the proximal and distal detector, respectively. L_{thr} indicates the path length corresponding to the RR weight threshold.

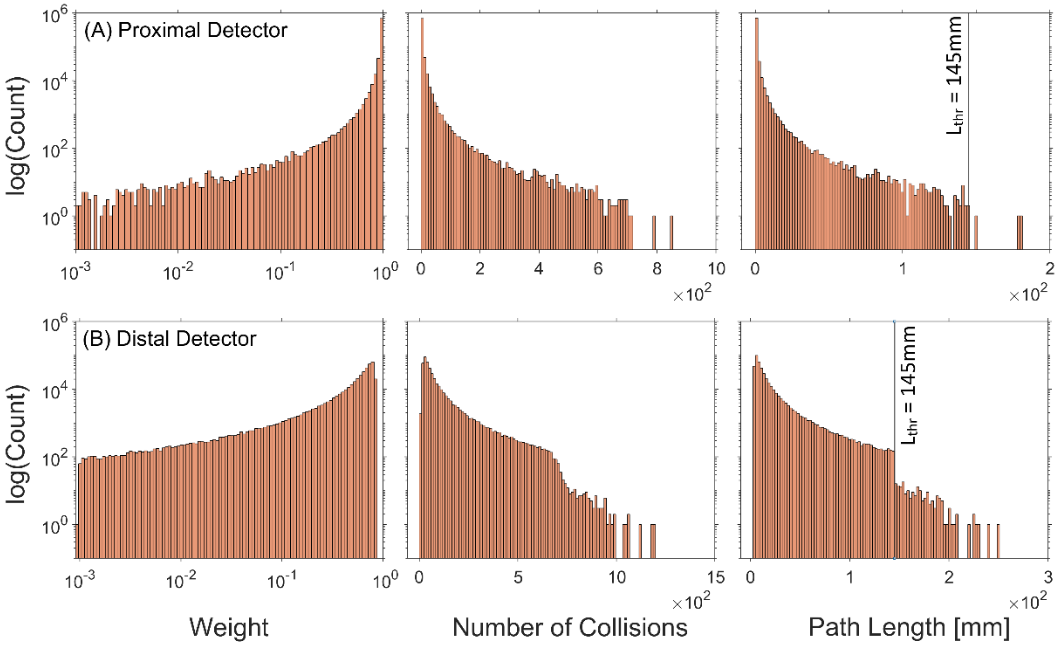

Figure D2. Histograms of W_R, *j* and *L* [mm] for tallied photons in the reference simulation performed at $\left(\mu_s'/\mu_a\right)$ = 20 using Russian Roulette. N_T = 782,075 and 402,520 for the proximal and distal detector, respectively. L_{thr} indicates the path length corresponding to the RR weight threshold.

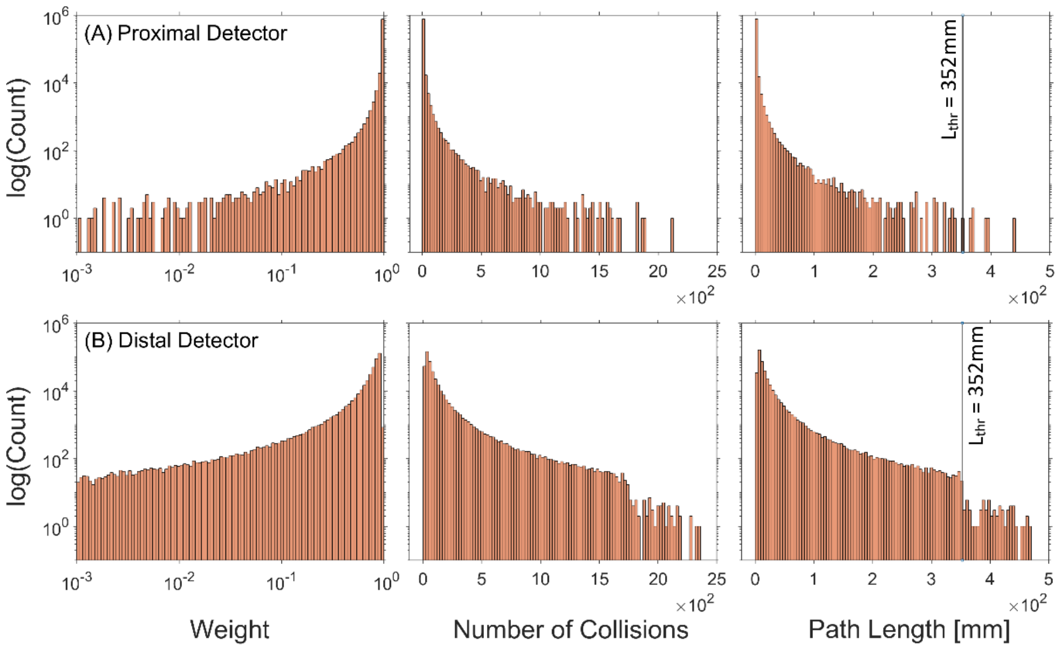

Figure D3. Histograms of W_R, j and L [mm] for tallied photons in the reference simulation performed at $\left(\mu_s'/\mu_a\right) =$ 50 using Russian Roulette. N_T = 808,170 and 405,654 for the proximal and distal detector, respectively. L_{thr} indicates the path length corresponding to the RR weight threshold.
